# Defect Engineering of Hexagonal MAB Phase Ti_2_InB_2_ as Anode of Lithium‐Ion Battery with Excellent Cycling Stability

**DOI:** 10.1002/advs.202308589

**Published:** 2024-03-15

**Authors:** Qing Shen, Yang Shi, Yibo He, Junjie Wang

**Affiliations:** ^1^ State Key Laboratory of Solidification Processing Northwestern Polytechnical University Xi'an Shaanxi 710072 P. R. China; ^2^ School of Materials Science and Engineering Northwestern Polytechnical University Xi'an Shaanxi 710072 P. R. China

**Keywords:** anode material, defect engineering, h‐MAB, lithium‐ion battery, Ti_2_InB_2_

## Abstract

Hexagonal MAB phases （*h*‐MAB） have attracted attention due to their potential to exfoliate into MBenes, similar to MXenes, which are predicted to be promising for Li‐ion battery applications. However, the high cost of synthesizing MBenes poses challenges for their use in batteries. This study presents a novel approach where a simple ball‐milling treatment is employed to enhance the purity of the *h*‐MAB phase Ti_2_InB_2_ and introduce significant indium defects, resulting in improved conductivity and the creation of abundant active sites. The synthesized Ti_2_InB_2_ with indium defects (V_In_‐Ti_2_InB_2_) exhibits excellent electrochemical properties, particularly exceptional long‐cycle stability at current densities of 5 A g^−1^ (5000 cycles, average capacity decay of 0.0018%) and 10 A g^−1^ (15 000 cycles, average capacity decay of 0.093%). The charge storage mechanism of V_In_‐Ti_2_InB_2_, involving a dual redox reaction, is proposed, where defects promote the In‐Li alloy reaction and a redox reaction with Li in the TiB layer. Finally, a Li‐ion full cell demonstrates cycling stability at 0.5 A g^−1^ after 350 cycles. This work presents the first accessible and scalable application of V_In_‐Ti_2_InB_2_ as a Li‐ion anode, unlocking a wealth of possibilities for sustainable electrochemical applications of *h*‐MAB phases.

## Graphical abstract

This work presents a simple and effective approach to introducing indium defects into the *h*‐MAB phase Ti_2_InB_2_, which exhibits exceptional long‐term cycling stability. This study highlights the novel functionality of *h*‐MAB phases in lithium‐ion batteries, shedding light on their potential applications in energy storage and beyond.

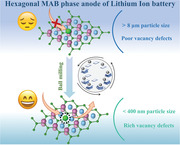

## 1 Introduction

Layered transition metal borides, known as MAB (M:transition metal, A:A group element, B:boron) phases, were discovered by Hillebrecht et.al.^[^ ^1^ ^]^ in 2015. These materials are considered the boron‐based counterparts of the well‐known MAX (M:transition metal, A:A group element, X:C or N) phases. It was initially believed that their 2D derivatives, called MBenes, could be obtained by selectively removing the A layers from MAB phases. However, the MAB phases proposed by Hillebrecht et al., possess orthorhombic symmetry (*ort*‐MAB), which differs from the hexagonal symmetry of MAX phases. As a result, they exhibit weak bonding anisotropy and poor exfoliability.^[^ ^2^ ^]^ Despite the extensive theoretical investigations carried out on the electrochemical properties of MBenes, the task of achieving a high‐quality exfoliation of the *ort*‐MAB phases remains a significant challenge. Recently, a new class of MAB phases with hexagonal symmetry (*h*‐MAB) has been predicted theoretically and successfully synthesized experimentally.^[^ ^3^, ^4^, ^5^ ^]^ For instance, in 2019, the first hexagonal ternary MAB phase Ti_2_InB_2_ was predicted and successfully synthesized;^[^ ^3^ ^]^ Subsequently, in 2020, Dirk Johrendt and Tobias Rackl synthesized Zr_2_SB and Hf_2_SB with a hexagonal structure via solid‐state reaction. Both compounds exhibit good metallic conductivity and Pauli paramagnetism.^[^ ^6^ ^]^ In 2021, Hu et al., reported the successful synthesis of hexagonal MAB phase Nb_2_SB ceramics with excellent mechanical properties and electrical conductivity using spark plasma sintering.^[^ ^7^ ^]^ Moreover, our group achieved the synthesis of a new *h*‐MAB phase, Hf_2_InB_2_, through a solid‐phase reaction in 2023 based on theoretical predictions.^[^ ^5^ ^]^ And the theoretical calculations indicated that the A layers of *h*‐MAB phases can be selectively removed with a high possibility, as their calculated separation energies are comparable to those of exfoliable MAX phases.^[^ ^3^, ^4^ ^]^ The theoretical predictions were confirmed through the experimental removal of In layers from the first synthesized *h*‐MAB Ti_2_InB_2_^[^ ^3^ ^]^ via de‐alloying and from *h*‐MAB Hf_2_InB_2_ through chemical etching.^[^ ^5^ ^]^ Moreover, recent theoretical calculations and experiments have provided evidence that the derived 2D MBenes (*h*‐MBenes) from *h*‐MAB exhibit great potential as anode materials in Li‐ion batteries (LIBs).^[^ ^3^, ^5^ ^]^

Anode materials play a crucial role in the electrochemical performance of LIBs. Currently, graphite is the most widely used commercial anode material for LIBs. However, its theoretical capacity is limited to only 372 mAh g^−1^, which hinders further improvement in battery performance.^[^ ^8^ ^]^ As a result, there has been a growing interest in exploring new anode materials with higher capacities. Researchers have focused on designing potential electrode materials such as MXenes and MBenes,^[^ ^9^, ^10^, ^11^, ^12^ ^]^ which exhibit excellent properties including excellent cycle stability and high rates. Nevertheless, to be commercially viable, these anode materials must also be cost‐effective. The high cost and low productivity associated with the chemical etching of MAX or MAB phases to obtain MXenes or MBenes raises uncertainties about their practical application. In comparison, MAX or MAB phases offer advantages in terms of preparation time and cost. It is regrettable that research on using MAX or MAB phases as battery electrodes is still in its very early stages. The Li‐storage performance of MAX phases has only been evaluated for a few compositions such as Ti_2_SC,^[^ ^13^, ^14^ ^]^ Ti_3_SiC_2_,^[^ ^15^ ^]^ Ti_3_Si_0.75_Al_0.25_C_2_,^[^ ^16^ ^]^ Ti_2_SnC,^[^ ^17^, ^18^ ^]^ V_2_SnC,^[^ ^19^ ^]^ and Nb_2_SnC.^[^ ^20^ ^]^ In 2019, Fokwa et.al.,^[^ ^21^ ^]^ reported that *ort*‐MAB phases Ni_2_ZnB and Ni_3_ZnB_2_ can be directly used as anodes for LIBs without the need for exfoliation, offering a new avenue for low‐cost applications of MAB phases. Unfortunately, the obtained capacities of Ni_2_ZnB (≈90 mAh g^−1^) and Ni_3_ZnB_2_ (≈70 mAh g^−1^) were much lower than that of commercial graphite (370 mAh g^−1^), making the *ort*‐MAB phase less attractive compared to other recently proposed anode materials. Recent studies have proven that the A atoms in *h*‐MAB phases exhibit higher activity compared to those in *ort*‐MAB phases.^[^ ^3^, ^4^, ^5^, ^22^ ^]^ This increased activity results in a greater number of A vacancies within the *h*‐MAB phase and it facilitates easier exposure of the MB faces when subjected to mechanical action. These findings strongly suggest that the *h*‐MAB phase could potentially possess superior properties as an anode material when compared to the *ort*‐MAB phase.

In this work, a defect engineering strategy was employed to enhance the electrochemical performance of the *h*‐MAB phase Ti_2_InB_2_. Initially, a simple post‐treatment utilizing ball‐milling was conducted to introduce a significant number of indium vacancies (V_In_) into the synthesized Ti_2_InB_2_ material due to its weak Ti‐In bonding. The resulting Ti_2_InB_2_ with rich In vacancies (referred to as V_In_‐Ti_2_InB_2_) was then utilized as an anode material for LIBs. The measured reversible capacities of the V_In_‐Ti_2_InB_2_ anodes were found to reach as high as 600 mA h g^−1^ at 100 mA g^−1^, exhibiting exceptional cycling stability even after 15 000 cycles at 10 A g^−1^. These results demonstrate a remarkable improvement in performance when compared to previously reported data on MAX and *ort*‐MAB phases. Furthermore, the analysis of CV measurement and pseudo‐capacitance behavior, in combination with density functional theory calculations, confirmed that the introduction of V_In_ contributes to an increase in active sites within the material. This, in turn, facilitates ion diffusion and electron transport, ultimately leading to enhanced charge and discharge capacities in the V_In_‐Ti_2_InB_2_ anodes.

## 2 Results and Discussion

### 2.1 Characterization of Synthesized Ti_2_InB_2_ Phase

**Figure 1:**
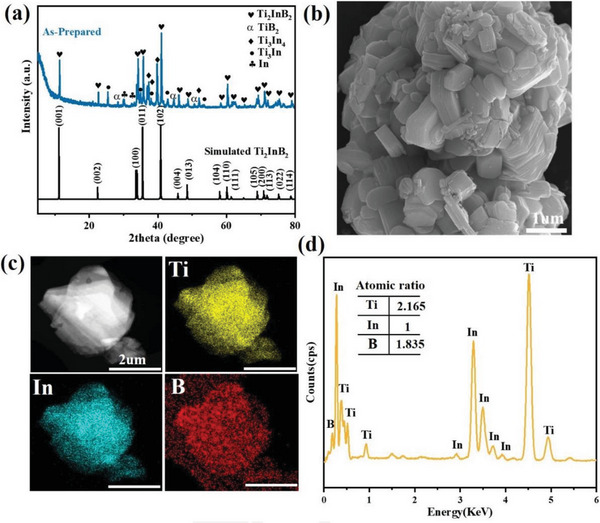
Characterization of synthesized *h*‐MAB phase Ti_2_InB_2_. a) The experimental measured and simulated X‐ray diffraction (XRD) patterns of as‐prepared Ti_2_InB_2_; b) SEM image and c) elemental mapping of Ti_2_InB_2_ sample; d) energy‐dispersive spectroscopy (EDS) spectrum of Ti_2_InB_2_.

The powder X‐ray diffraction (XRD) analysis, as shown in **Figure** **1**a, provided evidence of the crystalline phase and composition of the synthesized materials. The obtained XRD result confirms the successful synthesis of Ti_2_InB_2_ using a solid‐state reaction route. However, additional peaks at 2*θ* = 27.4°, 36.1°, 41.2°, 54.3°, and 56.6° corresponding to metal In, TiB_2_, Ti_3_In_4_, and Ti_3_In can also be observed. The presence of metallic phases, as well as mixtures and boride/carbide impurities, is an inevitable outcome during the synthesis of MAB/MAX phases.^[^ ^9^, ^23^ ^]^ It is worth noting that most of these impurities can be eliminated through chemical etching processes. The XRD patterns after HCl washing (refer to Figure S2, Supporting Information) reveal distinct diffraction peaks characteristic of the crystal structure of Ti_2_InB_2_ belonging to the hexagonal subgroup of *P6_3_*/*mmc* (No. 194). Specifically, diffraction peaks at 2*θ* = 11.1°, 22.4°, 33.6°, 35.6°, and 40.7° are attributed to crystal planes (001), (002), (100), (011), and (102), respectively. The persistent presence of TiB_2_ as a boride impurity often poses challenges to the synthesis of Ti‐based MAB phases.^[^ ^3^ ^]^ However, this study demonstrates that the effective removal of TiB_2_ impurities can be easily achieved through the addition of a simple ball milling process before hydrochloric acid cleaning. For more detailed information regarding the synthesis procedures, please refer to the Experimental Section. Figure S3 (Supporting Information) presents the optimized crystal structure model of Ti_2_InB_2_, highlighting its distinctive features. It exhibits a layered hexagonal structure (space group *P6m_2_* (No. 187)) composed of two M (Ti) layers and one A (In) layer arranged in an HCP A‐B‐A sequence. Remarkably, the sites between the M layers are occupied by boron atoms, forming a graphene‐like layer instead of an equilateral triangular plane. The graphene‐like layer consists of interconnected boron atoms arranged in a hexagonal pattern, which is known for its remarkable stability.^[^ ^4^ ^]^ The presence of this unique structure contributes to enhancing the cycling stability of batteries.^[^ ^9^, ^24^ ^]^

Here, Figure 1b displays a scanning electron microscopy (SEM) image of the bulk Ti_2_InB_2_ powders obtained. The image clearly shows the typical layered hexagonal structure of Ti_2_InB_2_.^[^ ^3^ ^]^ The powder particles, with a length and thickness of less than 1 µm, agglomerate in clusters of ≈8 µm. Previous studies have indicated that utilizing sub‐micrometer‐sized synthesized MAX/MAB phase thin flakes as anodes in lithium‐ion batteries (LIBs) could potentially improve their electrochemical performance.^[^ ^25^ ^]^ To examine the elemental distribution, energy‐dispersive X‐ray spectroscopy (EDS) elemental mapping was conducted on Ti_2_InB_2_, as depicted in Figure 1c. The mapping results indicate a homogeneous distribution of titanium (Ti), indium (In), and boron (B) elements within the material. Additionally, the EDS analysis shown in Figure 1d confirms the relative atomic composition of M_2_AB_2_ type MAB phases. Collectively, these findings provide solid confirmation regarding the crystal structure and elemental compositions of Ti_2_InB_2_ MAB phases.

### 2.2 Structure and Morphology of In‐Vacancy Defected Ti_2_InB_2_

**Figure 2:**
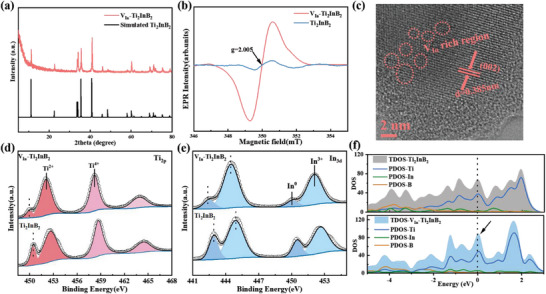
Characterization of V_In‐_Ti_2_InB_2_ treated by ball‐milling. a) The comparison of experimental measured and simulated X‐ray diffraction (XRD) patterns of V_In‐_Ti_2_InB_2_; b) Electron Paramagnetic Resonance (EPR) spectra of V_In‐_Ti_2_InB_2_ and pristine Ti_2_InB_2_; c) High‐Resolution Transmission Electron Microscopy (HRTEM) image of V_In‐_Ti_2_InB_2_; d) High‐resolution X‐ray Photoelectron Spectroscopy (XPS) spectra of Ti_2p_ and e) In_3d_ for V_In‐_Ti_2_InB_2_ and pristine Ti_2_InB_2_; f) Calculated Total Density of States (TDOS) and Partial Density of States (PDOS) for V_In‐_Ti_2_InB_2_ and Ti_2_InB_2_.

This subsection focuses on the investigation of the structure and morphology of In‐vacancy defects in Ti_2_InB_2_. The XRD pattern of V_In_‐Ti_2_InB_2_, obtained after ball‐milling and hydrochloric acid cleaning, is presented in **Figure** **2**a. Notably, no impurities were detected in the XRD pattern. This can be primarily attributed to the ball milling process, which reduces particle size, promotes chemical reactions, and facilitates the removal of impurities during the HCl cleaning process. Furthermore, the XRD peaks of V_In_‐Ti_2_InB_2_ appear lower compared to those of as‐synthesized and HCl‐etched Ti_2_InB_2_ samples (Figure S2, Supporting Information), indicating a smaller and thin‐layered stacking structure for V_In_‐Ti_2_InB_2_. The reduction in particle size following ball milling was confirmed through SEM imaging and particle size analyzer tests (Figures. S4 and S5, Supporting Information). Ball milling not only reduces the cluster size of Ti_2_InB_2_ from 8.41 µm to a single particle size of ≈0.37 um, but also induces the formation of In‐vacancy defects due to the mechanical forces exerted (Figure S5, Supporting Information). Figure S6 (Supporting Information) displays the increased specific surface area of the sample as a result of ball milling. According to Brunauer–Emmett–Teller theory calculations, the specific surface area of V_In_‐Ti_2_InB_2_ is higher than that of pristine Ti_2_InB_2_. This increase can be attributed to the production of small particles and thin layers, which expose more surface structure. Importantly, the enhanced specific surface area validates that the presence of point defects (in this case, indium vacancy defects) facilitates the adsorption of more active species and reactants on the surface.

To further examine the characteristics of vacancy features in the prepared samples, electron paramagnetic resonance (EPR) analysis was conducted. Figure 2b illustrates the EPR signal intensity at a g value of 2.005, which is associated with the concentration of indium vacancies.^[^ ^25^ ^]^ Furthermore, Figure 2c shows the observation of numerous In vacancies (circled regions), originating from the exfoliation of In atoms during the ball milling process under mechanical force. Moreover, the energy dispersive spectroscopy (EDS) spectra (Figure 1d; Figure S7, Supporting Information) of pristine Ti_2_InB_2_ and V_In_‐Ti_2_InB_2_ revealed that the element compositions were Ti_2_In_0.923_ and Ti_2_In_0.605_, respectively, implying that the In content of V_In_‐Ti_2_InB_2_ was lower than that of the In content in Ti_2_InB_2_. X‐ray photoelectron spectroscopy (XPS) measurements were also performed to investigate the presence of indium vacancies and the chemical information of V_In_‐Ti_2_InB_2_ and pristine Ti_2_InB_2_. The XPS full survey spectrum of V_In_‐Ti_2_InB_2_ and pristine Ti_2_InB_2_ clearly shows the signals corresponding to the Ti, In, and B elements (Figure S8a, Supporting Information). Detailed information regarding the elemental composition of the Ti_2_InB_2_ and V_In_‐Ti_2_InB_2_ samples, as measured by XPS analysis, can be found in Table S1 (Supporting Information). These results further confirm the presence of indium vacancies in V_In_‐Ti_2_InB_2_. The B 1s spectra exhibit two peaks at 191.7 and 186.6 eV, corresponding to the B─O and B─Ti, respectively (Figure S8b, Supporting Information).^[^ ^26^ ^]^ The binding energy of B 1s in V_In_‐Ti_2_InB_2_ also demonstrates a noticeable shift to lower energy, which we speculate is due to the increased electron concentration around V_In_ and the relatively higher electron concentration resulting from the strong electronegativity of B. Figure 2d,e displays the high‐resolution spectra of In 3d and Ti 2p in both Ti_2_InB_2_ and V_In_‐Ti_2_InB_2_. Specifically, for the Ti 2p, peaks corresponding to titanium within the Ti_2_InB_2_ lattice (Ti^2+^ at 449.9 and 452.1 eV) and titanium oxide/boride (Ti^4+^ at 458.2 and 463.8 eV) were observed in both Ti_2_InB_2_ and V_In_‐Ti_2_InB_2_ samples.^[^ ^26^, ^27^ ^]^ Similarly, for the In 3d, peaks representing indium within the Ti_2_InB_2_ lattice (In^0^ at 442.8 and 450.1 eV) and indium oxide (In^3+^ at 444.4 and 452.1 eV) were observed in both Ti_2_InB_2_ and V_In_‐Ti_2_InB_2_ samples.^[^ ^28^ ^]^ It is evident that after ball milling, the proportion of surface In^3+^ state significantly increases due to the expanded specific area, leading to greater exposure and oxidation of In atoms. Importantly, in comparison to pristine Ti_2_InB_2_, the binding energy of In_3_ *_d_* and Ti_2_ *_p_* in V_In_‐Ti_2_InB_2_ exhibited a noticeable shift toward lower energy due to the presence of defects in the V_In_‐Ti_2_InB_2_ structure.^[^ ^29^ ^]^ Thus, all these results provide strong evidence for the presence of indium vacancies in the synthesized material.

DFT calculations were performed to analyze the electronic structures of V_In_‐Ti_2_InB_2_ and Ti_2_InB_2_. The electron localization function and differential charge densities were used to visualize the effect of indium vacancies on electron rearrangement (Figure S9a–c, Supporting Information). In comparison to pristine Ti_2_InB_2_, V_In_‐Ti_2_InB_2_ exhibited an increase in electron concentration around the In vacancy. Figure 2f illustrates the projected density of states of different crystal orbitals for V_In_‐Ti_2_InB_2_ and Ti_2_InB_2_. The results indicated that V_In_‐Ti_2_InB_2_, as compared to Ti_2_InB_2_, displayed an electron increase phenomenon in the upper region of its valence band, resulting in a right shift of the Fermi energy. Additionally, V_In_‐Ti_2_InB_2_ exhibited higher occupied states across the Fermi level, suggesting enhanced conductivity. This can be attributed to the formation of In defects, which increased the contribution of In atoms to the states at the Fermi level. Therefore, V_In_‐Ti_2_InB_2_ exhibits enhanced metallic characteristics, contributing to improved electronic conductivity and, consequently, demonstrating excellent potential as an anode material.

### 2.3 Electrochemical Energy Storage Measurements

**Figure 3:**
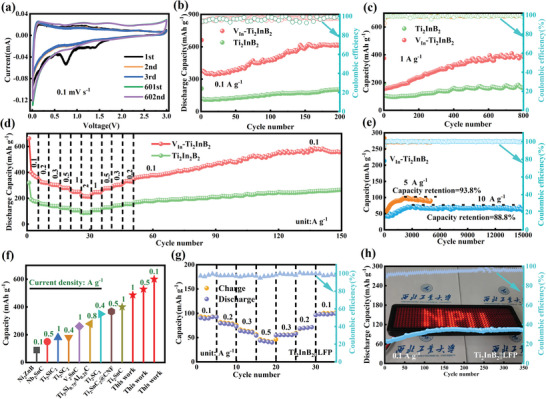
Charge–discharge properties for Ti_2_InB_2_‐based electrodes. a) CV curves of V_In_‐Ti_2_InB_2_ at a scan rate of 0.1 mV s^−1^; Cycling performance of Ti_2_InB_2_‐based electrodes at current densities of b) 0.1 A g^−1^ and c) 1 A g^−1^; d) Rate performance of Ti_2_InB_2_‐based electrodes; e) Long‐term cyclic performance of V_In_‐Ti_2_InB_2_ electrodes at current densities of 5 and 10 A g^−1^; f) Comparison of cyclic performance of V_In_‐Ti_2_InB_2_ electrode with other MAX or MAB anodes for LIBs; g) Rate capability and h) cycling performance of V_In_‐Ti_2_InB_2_||LFP full battery at a current density of 0.1 A g^−1^. The background of h) shows an LED powdered by one assembled V_In_‐Ti_2_InB_2_||LFP full battery.

To thoroughly investigate the Li^+^ storage performance of the Ti_2_InB_2_‐based electrodes, CR 2032 type coin cells were assembled using V_In_‐Ti_2_InB_2_ and pristine Ti_2_InB_2_ as the working anodes and Li foil as the counter electrode. **Figure** **3**a illustrates the cyclic voltammetry (CV) curves for the constructed V_In_‐Ti_2_InB_2_ electrodes at a scan rate of 0.1 mV s^−1^ within the voltage range of 0.01–3 V versus Li/Li^+^. The CVs of the first three cycles are compared with those of the 601 and 602 cycles (after cycling performance at a current density of 1 A g^−1^). It is observed that two irreversible peaks exist in the first cycle ≈0.75 and 1.5 V, which can be attributed to the irreversible contribution of the solid electrolyte interface (SEI) layer formation and electrolyte decomposition.^[^ ^16^ ^]^ Interestingly, small peaks at ≈0.7 and 0.01 V versus Li/Li^+^ appear in the oxidation and reduction curves, respectively, indicating the alloying reaction of In during cycling.^[^ ^30^ ^]^ To enhance the visibility of these peaks, a special half‐cell Li‐ion battery was assembled using pure V_In_‐Ti_2_InB_2_ powder as the working electrode without any conductive additives, copper foil, or binder (Figure S10, Supporting Information). Figure S11a (Supporting Information) demonstrates the CV curves of pure V_In_‐Ti_2_InB_2_ powder, where the presence of alloy LiIn (0.01 V, 0.72 V) confirms the occurrence of the alloying reaction.^[^ ^30^, ^31^ ^]^ Another broad oxidation peak at ≈1.6 V and a redox peak at 0.9 V may be attributed to the redox reaction between Li^+^ and the MAB phase.^[^ ^32^ ^]^ Conversely, the CV curves of pristine Ti_2_InB_2_ (Figure S11b, Supporting Information) do not exhibit obvious peaks related to the alloying reaction of In. This stark difference further supports the notion that ball milling exposes more In atoms and promotes the occurrence of alloying reactions, thereby increasing the Li^+^ storage capacity. Furthermore, comparing the CV curves of the 601 and 602 cycles with the first three cycles (Figure 3a), although no new redox peaks emerge or disappear, the overall shape of the curve undergoes significant changes to pseudocapacitive‐shaped CV curves after 600 cycles. This change facilitates rapid Li^+^ migration in the V_In_‐Ti_2_InB_2_ anode.^[^ ^33^ ^]^

Figure 3b presents the short‐term cycling performance of pristine Ti_2_InB_2_ and V_In_‐Ti_2_InB_2_ electrodes at a current density of 0.1 A g^−1^. The first discharge‐specific capacities of V_In_‐Ti_2_InB_2_ and pristine Ti_2_InB_2_ were measured to be 663 mAh g^−1^ and 213 mAh g^−1^, respectively, while the second discharge‐specific capacities dropped to 371 and 125 mAh g^−1^, respectively. The initial capacity loss in the first cycle can be attributed to the irreversible formation of a solid electrolyte interface.^[^ ^34^ ^]^ Remarkably, the specific capacity of the V_In_‐Ti_2_InB_2_ electrode exhibited a gradual increase with cycling, reaching a stable reversible specific capacity of 600 mAh g^−1^ after 150 cycles at 0.1 A g^−1^, which remained stable up to 200 cycles. In contrast, pristine Ti_2_InB_2_ only achieved a stable reversible specific capacity of 200 mAh g^−1^. The experimental results are consistent with the calculated theoretical capacities. Specifically, the calculated theoretical capacity of V_In_‐Ti_2_InB_2_ (716.97 mAh g^−1^) is higher than that of the Ti_2_InB_2_ monolayer (586.5 mAh g^−1^) (Figure S12, Supporting Information). The initial coulombic efficiency of V_In_‐Ti_2_InB_2_ and pristine Ti_2_InB_2_ is 55.9% and 54.54%, respectively. However, it increased to ≈100% after several cycles and remained stable in subsequent cycles (Figure S13, Supporting Information). This result is consistent with the discharge/charge curves, which show nearly overlapping behavior from cycles 150–200 (Figure S13a, Supporting Information). The discharge/charge curves of pristine Ti_2_InB_2_ are shown in Figure S13b (Supporting Information). Both pristine Ti_2_InB_2_ and V_In_‐Ti_2_InB_2_ electrodes exhibit capacity‐uplifting behavior, which is mainly attributed to the increased interlayer spacing of the *h*‐MAB phase after repeated electrochemical etching reactions. This leads to more inattentive particles and improved electrolyte infiltration, thereby promoting more electrochemical active sites for Li^+^ insertion and alloying. The detailed mechanism will be discussed later and combined with other results. Additionally, it is worth noting that the capacity‐uplifting behavior was commonly observed in MAX‐based anode materials.^[^ ^14^, ^20^ ^]^ Furthermore, at a current density of 1 A g^−1^, the V_In_‐Ti_2_InB_2_ anode continued to demonstrate capacity enhancement, achieving a high stable discharge capacity of ≈400 mAh g^−1^ after 600 cycles, which was sustained up to 800 cycles and significantly higher than that of pristine Ti_2_InB_2_ (170 mAh g^−1^) (Figure 3c). This highlights the importance of decreasing particle size and inducing defects to achieve high electrochemical performance. Furthermore, the rate performance of both electrodes was evaluated under various current densities ranging from 0.1 to 2 A g^−1^. The V_In_‐Ti_2_InB_2_ anode exhibits reversible capacities of 384, 322, 304, 281, 249, and 217 mAh g^−1^ at current densities of 0.1, 0.2, 0.3, 0.5, 1, and 2 A g^−1^, respectively. In contrast, the pristine Ti_2_InB_2_ electrode only achieves reversible capacities of 180, 155, 141, 119, 105, and 87 mAh g^−1^ under the corresponding current densities. Notably, when the current density returns to 0.1 A g^−1^, the discharge capacity of V_In_‐Ti_2_InB_2_ increases from 369 to 600 mAh g^−1^ after 150 cycles (Figure 3d). Moreover, the rate performance further improves after 150 cycles, as shown in Figure S14a (Supporting Information). At the same current densities of 0.1 to 2 A g^−1^, the capacities increase to 600, 581, 531, 528, 483, and 410 mAh g^−1^, respectively, which are nearly double the initial values. Furthermore, the long‐term cycling performance of the V_In_‐Ti_2_InB_2_ electrode was tested under high current conditions. Impressively, Figure 3e demonstrates high retention ratios of 93.8% and 88.8% for the 5000 and 15 000 cycle capacities, respectively, at high current densities of 5 and 10 A g^−1^. While at high current densities of 3 and 5 A g^−1^, the Ti_2_InB_2_ electrode exhibited lower capacities of 50 mAh g^−1^ after 5000 cycles and 40 mAh g^−1^ after 15 000 cycles (Figure S14b, Supporting Information). Additionally, the Coulombic efficiency of the V_In_‐Ti_2_InB_2_ anode remains ≈100% throughout the entire cycling process, except for the initial irreversible capacity loss associated with the formation of a solid electrolyte interface (SEI) film.^[^ ^28^ ^]^ The exceptional long‐term cyclic stability of the V_In_‐Ti_2_InB_2_ electrode can be attributed mainly to the stable hexagonal layer structure of boron atoms, which enhances its volume buffering capability. Importantly, the electrochemical performance of the V_In_‐Ti_2_InB_2_ electrode is currently the best among all reported MAX/MAB‐based anodes for lithium‐ion batteries (Figure 3f; Table S2, Supporting Information).

The remarkable lithium storage performance of the V_In_‐Ti_2_InB_2_ electrode in half cells has sparked interest in exploring its potential application in full battery systems. These systems involve pairing a V_In_‐Ti_2_InB_2_ anode with a LiFePO_4_ (LFP) cathode. The XRD pattern and morphology of the LFP powders are depicted in Figure S15 (Supporting Information), while Figure S16 (Supporting Information) demonstrates that the LFP cathode exhibits a discharge/charge plateau at 3.5/3.36 V and a reversible capacity of 150 mAh g^−1^ at a current rate of 100 mA g^−1^ in the half cell. To prevent the loss of limited Li ions in the full battery configuration, the V_In_‐Ti_2_InB_2_ anode undergoes three precycles to establish a stable solid electrolyte interface (SEI) protective film in the half cell. The rate performance of the V_In_‐Ti_2_InB_2_||LFP full battery is presented in Figure 3g. The full battery demonstrates discharge capacities of 92, 78, 64, and 44 mAh g^−1^ at current rates of 100, 200, 300, and 500 mA g^−1^, respectively. Notably, when the current density was reduced back to 100 mA g^−1^, the capacity could be restored to its initial level, indicating excellent structural integrity of the full cell. Furthermore, the full battery exhibits a remarkable discharge capacity of 106 mAh g^−1^ after 350 cycles at 0.1 A g^−1^ (Figure 3h). This capacity rise phenomenon in the full cell system can also be attributed to the earlier mentioned pillaring effect. Additionally, to demonstrate the practical application of the constructed full battery, an experiment was conducted using a single‐button battery to successfully illuminate red light‐emitting diode (LED) panels displaying the letters “NPU.” These results collectively highlight the significant potential of the synthesized V_In_‐Ti_2_InB_2_ as an anode material for achieving outstanding performance in lithium‐ion batteries.

### 2.4 Ultrafast Ion Transportation Kinetic Analysis of V_In_‐Ti_2_InB_2_ Electrode

**Figure 4:**
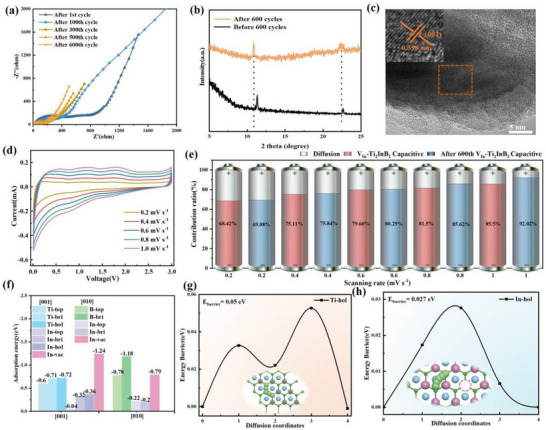
Mechanism study for Li storage and diffusion on the V_In_‐Ti_2_InB_2_ electrode. a) EIS measurements of the V_In_‐Ti_2_InB_2_ electrode after different cycles; b) XRD pattern and c) HRTEM images of the V_In_‐Ti_2_InB_2_ electrode after 600 cycles; d) CV curves of the V_In_‐Ti_2_InB_2_ electrode with different scan rates after 600 cycles; e) Capacitive contribution ratio of the V_In_‐Ti_2_InB_2_ electrode at different rates before and after 600 cycles; f) Calculated adsorption energies of Li atoms at different sites on the V_In_‐Ti_2_InB_2_ (001) and (010) surfaces; Calculated energy barriers for Li migration along the paths of g) Ti‐hol to Ti‐hol and h) In‐hol to In‐hol on the (001) surfaces.

The reaction kinetics of Li‐ion electrodes can be probed by the electrochemical impedance spectroscopy (EIS) tests^[^ ^35^ ^]^ and galvanostatic intermittent titration technique (GITT).^[^ ^36^ ^]^ **Figures** **4**a and S17a (Supporting Information) display the Nyquist plots, which exhibit a depressed semicircle in the high‐frequency region representing the internal resistance, including the resistance within the active materials and the contact resistance between the current collector and the active materials.^[^ ^37^ ^]^ Additionally, an inclined line is observed in the low‐frequency region, indicating the Warburg resistance between the electrodes and electrolytes.^[^ ^38^ ^]^ In Figures 4a and S17a (Supporting Information), it is observed that the V_In_‐Ti_2_InB_2_ electrode exhibits a smaller semi‐arc radius than the pristine Ti_2_InB_2_ electrode at high frequencies, indicating lower charge transport resistance. Furthermore, with an increasing number of cycles, the semi‐circular arc radius of both the V_In_‐Ti_2_InB_2_ and pristine Ti_2_InB_2_ electrodes progressively decreases. As the number of cycles increases, the radius of the semicircular arc of the V_In_‐Ti_2_InB_2_ electrode decreases progressively. This reduction can be attributed to the enhanced transfer of ions and electrons within the electrode, indicating the self‐improvement of the electrodes and the rapid transfer of electrons during the electrochemical reaction. This phenomenon is responsible for the observed improvement in capacity. Furthermore, the diffusion kinetics of the V_In_‐Ti_2_InB_2_ electrode were investigated using GITT.^[^ ^38^ ^]^ Figure S17b (Supporting Information) illustrates the GITT profiles for both the lithiation and delithiation processes based on the potential‐time response plot. The Li^+^ diffusion coefficient (*D*_Li_) was calculated using the GITT equation.^[^ ^38^ ^]^ The extrapolated value of *D*_Li_ was determined to be 4.7  ×  10^−11^ cm^2^ s^−1^, surpassing that of commercial graphite (4.0  ×  10^−11^cm^2^ s^−1^),^[^ ^39^ ^]^ Li_4_Ti_5_O_12_ (4.0  ×  10^−15^cm^2^ s^−1^)^[^ ^40^ ^]^ and Si (4.0  ×  10^−14^cm^2^ s^−1^).^[^ ^41^ ^]^ This result demonstrates that V_In_‐Ti_2_InB_2_ exhibits a higher Li^+^ diffusion coefficient, affirming its potential as a promising anode material.

Furthermore, to gain a comprehensive understanding of the high capacity and charge storage mechanism of the V_In_‐Ti_2_InB_2_ anode, we conducted ex situ XRD and XPS characterizations during the charge–discharge process, followed by SEM, TEM, and HRTEM characterizations after 600 cycles at 1 A g^−1^. The ex situ XPS characterization results of the V_In_‐Ti_2_InB_2_ anode at pristine, lithiated, and delithiated states are presented in Figure S18 (Supporting Information). The In 3d spectra exhibited weakened peaks at 442.5, 444.5, 450.1, and 451.9 eV upon discharge to 0.01 V, which reappeared when charged to 3 V, indicating the reversibility of the alloying/dealloying process of the A‐site Indium(Figure S18a, Supporting Information).^[^ ^28^, ^42^ ^]^ Additionally, the C 1s spectra revealed the formation of the SEI film after charge and discharge (Figure S18b, Supporting Information).^[^ ^42^ ^]^ However, after 600 cycles, only the peaks for indium oxide (In^3+^, 444.4 and 452.1 eV) were observed in the In 3d spectra, suggesting significant exfoliation of In from the MAB phase due to lithiation/delithiation cycles.^[^ ^20^ ^]^ The Ti 2p and B 1s of the V_In_‐Ti_2_InB_2_ anode before and after cycling exhibited similar binding energy, indicating the chemical stability of the V_In_‐Ti_2_InB_2_ electrode (Figure S19, Supporting Information). Ex situ‐XRD investigations of V_In_‐Ti_2_InB_2_ electrodes during charge/discharge processes were performed. As shown in Figure S20a (Supporting Information), the XRD diffraction peaks of V_In_‐Ti_2_InB_2_ did not exhibit noticeable changes at different voltages, indicating the stable crystal structure of V_In_‐Ti_2_InB_2_ without phase transition or evolution of lattice constant. Notably, the XRD pattern of the sample after 600 cycles (Figure 4b; Figure S20b, Supporting Information) revealed a significant leftward peak shift and broadening of the (001) and (002) peaks, further indicating that the V_In_‐Ti_2_InB_2_ MAB phase can be exfoliated by lithiation/delithiation cycles, leading to size reduction, which is consistent with the TEM and HRTEM images. As shown in Figure 4c, the HRTEM image reveals a larger interlayer spacing of ≈0.398 nm in V_In_‐Ti_2_InB_2_ electrode after 600 cycles. It suggests that the interlayer spacing expands and the particle size decreases during the lithiation and delithiation processes.

Furthermore, the cross‐section and top views of SEM images of the V_In_‐Ti_2_InB_2_ anode after 600 cycles are displayed in Figures S21 and S22 (Supporting Information). Figure S21 (Supporting Information) shows a substantial increase in volume compared to the initial state, while Figure S22 (Supporting Information) demonstrates uniform particle size and a stable structure, contributing to the achievement of long cycling stability. Moreover, the TEM image in Figure S23 (Supporting Information) exhibits thin and even monolayer nanosheets. All of these observations indicate that the expansion of the V_In_‐Ti_2_InB_2_ anode is likely due to the penetration of lithium ions into the interlayers and repeated alloying reactions between In and Li. As the particle size decreases and the interlayer spacing expands, it is anticipated that the contribution of EDLC/pseudocapacitance will gradually increase in subsequent cycles. This conclusion is further supported by the subsequent pseudocapacitance analysis.

To gain a deeper understanding of the exceptional lithium storage kinetics of the V_In_‐Ti_2_InB_2_ electrode, cyclic voltammetry (CV) measurements were conducted at different scan rates for both the pristine electrode and after 600 cycles. The characteristic peaks exhibited similar shapes as the scan rates increased (Figure S24a, Supporting Information). However, after 600 cycles, the CV curves showed a more rectangular shape, with a wider redox peak (Figure 4d), suggesting a transition toward a capacitive mechanism. This phenomenon could be one of the factors contributing to the observed increase in capacity. Furthermore, the charge storage mechanism was analyzed by examining the relationship between the peak current (*i*) and the scan rate (*ν*), which can be calculated using the following equation:^[^ ^43^, ^44^, ^45^ ^]^

(1) $$i\;\left(v\right)=\;a\times v^{b}$$

where *a* and *b* are adjustable parameters. The value of “*b*” can be obtained by fitting the log (*i*) versus log (*ν*) data. Generally, “*b*” ranges from 0.5 to 1.0, indicating three possible scenarios: 1) When “*b*” equals 0.5, it suggests a diffusion‐controlled charge storage mechanism; 2) When “*b*” equals 1.0, it indicates a capacitive charge storage mechanism; 3) When “*b*” falls between 0.5 and 1.0, it implies a combined contribution from both capacitance and diffusion control mechanisms. As depicted in Figure S25a (Supporting Information), the calculated values of “*b*” were determined to be 0.62 for the cathodic peak and 0.76 for the anodic peak, suggesting the Li^+^ storage behavior in V_In_‐Ti_2_InB_2_ is controlled collectively by ionic diffusion and capacitive processes, which leads to fast Li^+^ diffusion kinetics enabling the high‐rate performance. Additionally, the ratios of the capacitive contribution (*k*_1_ × *ν*) and the diffusion‐controlled contribution (*k*_2_ × *ν* ^1/2^) can be calculated using the following equation:^[^ ^46^, ^47^ ^]^

(2) $$i\left(v\right)\;=k_{1}\;\times v+k_{2}\times v^{1/2}$$

As shown in Figure S25b (Supporting Information), the capacitive contribution to the total capacity of V_In_‐Ti_2_InB_2_ anode is 80.29% at a sweep rate of 0.6 mV s^−1^. Additionally, the precycling V_In_‐Ti_2_InB_2_ electrode exhibits similar pseudocapacitive behavior (see Figure S24, Supporting Information). Figure 4e presents a summary of the pseudocapacitive contribution ratios for the V_In_‐Ti_2_InB_2_ electrode before and after 600 cycles at various rates. The results indicate that the pseudocapacitive contribution ratio gradually improves with increasing scan rate. Moreover, after cycling for the 600 times, the V_In_‐Ti_2_InB_2_ electrode shows a higher pseudocapacitive ratio compared to the precycling V_In_‐Ti_2_InB_2_ electrode at the same sweep rate. Furthermore, we also conducted a similar analysis on the storage mechanism of the pristine Ti_2_InB_2_ electrode (Figure S26, Supporting Information). By comparing the precycling of the V_In_‐Ti_2_InB_2_ and pristine Ti_2_InB_2_ electrodes, we obtained a similar result, indicating that the pseudocapacitive ratio of the V_In_‐Ti_2_InB_2_ electrode is higher than that of the Ti_2_InB_2_ electrode at the same scanning rate. This result further provides evidence that the presence of indium defects is a crucial factor for achieving the high electrochemical performance of V_In_‐Ti_2_InB_2_.

The ball‐milling treatment can significantly reduce the particle size of Ti_2_InB_2_. First, the Ti_2_InB_2_ crystal can be fractured along the (001) plane, which can produce Ti‐ and In‐terminated (001) surfaces. At the same time, ball‐milling treatment will also increase the exposure probability of (010) end face. To evaluate the impact of defects and analyze the charge storage mechanism of V_In_‐Ti_2_InB_2_, we conducted DFT calculations to investigate the adsorption of Li atoms on Ti_2_InB_2_ and V_In_‐Ti_2_InB_2_ (001) and (010) surfaces, considering various Ti, In, and B atom configurations. Our study considered potential active adsorption sites such as top, bridge (bri), hollow (hol), and vacancy (vac) for the adsorbed lithium atom on Ti‐, In‐ and B‐terminated surfaces (see details of the possible adsorption configurations in Figure S27, Supporting Information). Figures 4f and S28a (Supporting Information) summarize the calculated adsorption energies for Li atoms at different sites on various surface models of V_In_‐Ti_2_InB_2_ and pristine Ti_2_InB_2_, respectively. It is evident that the adsorption strength of Li atoms on Ti‐terminated (001) surfaces (−0.6 to −0.72 eV) is significantly greater than that on In‐terminated (001) surfaces (−0.04 to −0.36 eV). However, the adsorption of Li atoms on In‐vacancy‐containing In‐terminated (001) surfaces (−1.24 eV) becomes even stronger than that on Ti‐terminated (001) surfaces. The results indicate that the introduction of an indium defect enhances the adsorption of lithium atoms at (001) surface.

Calculation results show that the adsorption of Li at V_In_‐Ti_2_InB_2_ (010) surface is quite strong compared with those at (001) surface. The most negative adsorption energies at B and In sites of (010) surface are −1.18 and −0.79 eV (Figure 4f), respectively. Therefore, one can conclude that strong Li‐B and Li‐In alloy reactions happen at V_In_‐Ti_2_InB_2_ (010) end during the charge/discharge process of V_In_‐Ti_2_InB_2_ anode. Especially, In vacancy at the (010) end can dramatically enhance the Li‐In interaction, which is proven by the change of adsorption energy from −0.2 eV to −0.79 eV. Furthermore, the charge density difference also indicates a significant degree of charge transfer between Li and the B site, as well as between Li and the In vacancy in the V_In_‐Ti_2_InB_2_ (Figures S28b and S29, Supporting Information). These findings suggest that the charge transfer capability between lithium and the vacancy is notably enhanced for V_In_‐Ti_2_InB_2_, which would potentially enhance the charge transfer kinetics when utilized as a battery electrode. Consequently, besides improving the intrinsic electron conductivity of Ti_2_InB_2_, the presence of an indium vacancy can also provide more active sites for robust Li‐ion adsorption. This ultimately leads to a higher capacity of V_In_‐Ti_2_InB_2_ as an anode material. Importantly, these findings are consistent with experimental observations (Figure S11a, Supporting Information).

Furthermore, it was found that the substantial position preference energy not only impacts the specific capacity of the material but also influences its diffusion kinetics. The CI‐NEB calculation provided valuable insights into the diffusion barriers of Li atoms in different sites. On the Ti‐terminated (001) surface (Figure 4g), the calculated diffusion barrier between neighboring Ti‐hol sites was found to be 0.05 eV. Similarly, on the (010) surface (Figure S30a, Supporting Information), the diffusion barrier between neighboring B‐bri sites was determined to be 0.35 eV. These values are comparable to diffusion barriers observed in other commonly used anodes for LIBs.^[^ ^48^, ^49^ ^]^ Additionally, significantly lower energy barriers of 0.027 and 0.018 eV were obtained for Li diffusion between neighboring In‐hol sites on the In‐terminated (001) surface and In‐top sites on the In‐terminated (010) surface of V_In_‐Ti_2_InB_2_, respectively (Figure 4h; Figure S30b, Supporting Information).

**Figure 5:**
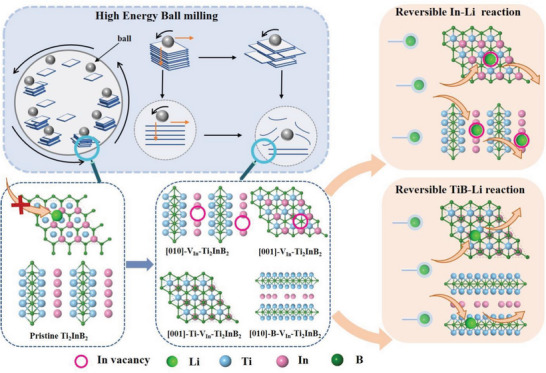
The schematic illustration of the energy storage mechanism in the V_In_‐Ti_2_InB_2_ cells.

Finally, a dual energy storage mechanism was proposed to explain the superior lithium storage performance of V_In_‐Ti_2_InB_2_ compared to pristine Ti_2_InB_2_ (**Figure** **5**), integrating theoretical calculations and experimental tests. First, the simple ball‐milling treatment employed in this study significantly increased the exposure of (001) and (010) planes, resulting in a substantial number of In vacancies. Then, during the charge/discharge process of the V_In_‐Ti_2_InB_2_ anode, two types of reactions occurred: defect‐mediated alloy reactions at In sites on the (010) end and (001) surfaces, and redox reactions at B sites on the (010) end and Ti‐terminated (001) surface, as depicted in Figure 5. Additionally, previous studies have demonstrated that the separation energy of the Ti_2_InB_2_ (001) surface is lower than that of the (010) surface.^[^ ^3^ ^]^ Therefore, it is expected that the ball‐milling treatment will predominantly expose the (001) plane in the treated Ti_2_InB_2_ sample. This suggests that the presence of indium defects on the (001) surface of Ti_2_InB_2_ plays a crucial role in modifying the electronic structure and facilitating faster Li transport processes, thereby ensuring stable cyclability and high‐rate performance of the V_In_‐Ti_2_InB_2_ anode. Consequently, this contributes to achieving high‐rate performance in Li‐ion batteries. Therefore, the presence of indium defects not only enhances the capacity for Li^+^ ions but also improves the kinetics of Li+ ion diffusion in V_In_‐Ti_2_InB_2_. This finding underscores the significance of defect engineering in optimizing both electronic properties and Li+ ion transport, thereby enabling the realization of Li‐ion batteries with excellent high‐rate performance.

## 3 Conclusion

In summary, this work presents a simple but very effective approach to introducing indium defects into the *h*‐MAB phase Ti_2_InB_2_ via a ball milling method. Remarkably, the indium‐defected Ti_2_InB_2_ MAB phase (V_In_‐Ti_2_InB_2_) as an anode for LIBs achieved a maximum lithium storage capacity of up to 600 mAh g^−1^, surpassing the highest capacities reported in the literature for similar MAX/MAB phase anodes. The outstanding capacity obtained through this method is attributed to the following factors. First, the ball milling process reduces the particle size of the *h*‐MAB phase, leading to increased surface area and exposure of more indium atoms, which promotes the reaction between lithium ions (Li^+^) and indium atoms, resulting in the formation of a Li‐In alloy and contributing to the enhanced capacity. Second, the introduction of indium defects in the structure of Ti_2_InB_2_ reduces the diffusion energy barrier of lithium ions, facilitating the interfacial electron/mass transfer and enhancing the rate performance. Moreover, benefiting from the structural advantages of the *h*‐MAB phase, the V_In_‐Ti_2_InB_2_ electrode exhibits exceptional long‐term cycling stability. Based on these findings, this research opens new avenues for improving the performance and stability of alloying materials and encourages further investigations into the potential applications of other *h*‐MAB family members in various electrochemical systems.

## Conflict of Interest

The authors declare no conflict of interest.

## Supporting information

Supporting Information

## Data Availability

The data that support the findings of this study are available from the corresponding author upon reasonable request.
