# Supplementary material for: Defect Engineering of Hexagonal MAB Phase Ti2InB2 as Anode of Lithium‐Ion Battery with Excellent Cycling Stability
Source: Adv Sci (Weinh). 2024 Mar 15;11(21):2308589. doi: 10.1002/advs.202308589 (PMC11151021; doi:10.1002/advs.202308589)
Supplement: Supplementary file 1 — Supporting Information [file ADVS-11-2308589-s001.pdf]

ADVANCED SCIENCE
Open Access

Supporting Information

for *Adv. Sci.*, DOI 10.1002/advs.202308589

Defect Engineering of Hexagonal MAB Phase Ti_2InB_2 as Anode of Lithium-Ion Battery with Excellent Cycling Stability

*Qing Shen, Yang Shi, Yibo He and Junjie Wang**

Supporting information

Defect Engineering of Hexagonal MAB Phase Ti_2InB_2 as Anode of Lithium-ion Battery with Excellent Cycling Stability

Qing Shen,[a,b] Yang Shi, [b] Yibo He, [a,b] and Junjie Wang [a,b]*

a. State Key Laboratory of Solidification Processing, Northwestern Polytechnical University, Xi' an, Shaanxi 710072, People's Republic of China.

b. School of Materials Science and Engineering, Northwestern Polytechnical University, Xi'an, Shaanxi 710072, People's Republic of China.

* Corresponding author.

E-mail address: wang.junjie@nwpu.edu.cn.

Preparation of V_{In}-Ti_2InB_2 MAB phase

According to the previous report[1], titanium powder (~ 300 mesh, 99.5 wt% purity), indium powder (~ 300 mesh, 99.5 wt% purity), and boron powder (~ 300 mesh, 99.5 wt% purity) were mixed in a Ti:In:B molar ratio of 2.5:1.5:2 using mixing oscillations for ten minutes. Subsequently, the precursor powder mixture was wrapped in Mo foil and placed inside an aluminum oxide boat. The boat was then transferred to a tube furnace and heated to 1100 °C for 24 hours at a heating rate of 5 °C min^{-1} under an argon atmosphere. After the completion of the reaction, the resulting bulk Ti_2InB_2 material was placed in a stainless-steel container and subjected to ball milling for 5 hours at a speed of 300 rpm. It was then stirred with HCl solution (2 mol L^{-1}, 100 mL) at room temperature for 24 hours, leading to the formation of Indium vacancies in Ti_2InB_2 (V_{In}-Ti_2InB_2). The pristine Ti_2InB_2 sample was prepared using the same method without the ball-milling procedure.

Characterizations and Measurements

An X-ray diffractometer (XRD, DX-2700B) operating at 40 kV and 200 mA with Cu Kα radiation (λ = 0.15406 nm) was utilized to characterize the compositions of the samples. Scanning electron microscopy (SEM, JSM-6700F), transmission electron microscopy (TEM), and high-resolution transmission electron microscopy (HRTEM, JEM-2200FS) were employed for observing

the morphology and microstructure of the samples. The instruments were also equipped with energy-dispersive X-ray spectroscopy (EDS) for elemental mapping. X-ray photoelectron spectroscopy (XPS, ESCALAB) analysis was carried out using an Mg-Kα light source. Electron paramagnetic resonance (EPR, Bruker EMXnano) was utilized to detect unpaired electrons present in atoms or molecules within the material.

Electrochemical Tests

CR-2032 type coin-cells were assembled inside an argon-filled glove box with H_2O and O_2 concentrations kept below 0.1 ppm. For electrode preparation, a slurry was formed by uniformly grinding 70 wt% active material, 20 wt% carbon black, and 10 wt% polyvinylidene fluoride (PVDF) in 1-methyl-2-pyrrolidone (NMP). This slurry was then coated onto copper foil. After vacuum drying at 60 °C for 24 hours, circular electrodes with a diameter of 12 mm and an average mass of active materials approximately 1.0 mg cm^{-2} were obtained from the electrode tape. The counter electrode, electrolyte, and separator used were lithium foil, 1 M $LiPF_6$ (ethylene carbonate (EC): diethyl carbonate (DEC) = 1:1 v/v), and Cellgard 2400, respectively.

Galvanostatic charge/discharge (GCD) measurements and Galvanostatic Intermittent Titration Technique (GITT) were performed using the Neware battery test system (Shenzhen, China) at various current densities within a voltage range of 0.01–3.0 V. Electrochemical impedance spectroscopy (EIS) from 0.01 Hz to 100 kHz and Cyclic voltammetry (CV) were conducted using an electrochemical workstation (CHI660E). Furthermore, a lithium-ion full cell was constructed with $LiFePO_4$ (LFP) as the cathode and V_{In}-Ti_2InB_2 as the anode. The LFP powders were purchased from Shenzhen KeLuDe Technology for the assembly of the V_{In}-Ti_2InB_2||LFP full battery. The cathode was prepared by mixing active material (80%), carbon black (10%), and PVDF (10%) to form a homogeneous slurry in NMP, which was then coated on Al foil. The separator and electrolyte used were consistent with those in half cells. To establish a stable solid electrolyte interface layer, the V_{In}-Ti_2InB_2 anode was activated at 0.05 A g^{-1} for 3 cycles in half cells. Additionally, the mass ratio of anode to cathode (N/P) was maintained at 1:1.2, and the specific capacities of the full-cell devices were determined based on the mass of the anode. Considering the potential difference between the cathode and anode, the voltage range for the GCD tests in the full battery was set to 0.5-4.0 V.

Calculation Setting

The Vienna Ab-initio Simulation Package (VASP)[2] was utilized in this study for all density functional theory (DFT)-based calculations. The calculations involved the use of the projector augmented plane-wave (PAW)[3] method and the Perdew–Burke–Ernzerhof (PBE)[4] functional within the generalized gradient approximation (GGA) to compute the exchange-correlation interaction energy. For the relaxation and total energy calculations of the slab models, a Monkhorst-Pack k-point mesh with a sampling of 4×4×1 was employed. The cutoff energy of the plane wave basis was set to 520 eV to ensure accurate results. Furthermore, the energy convergence criterion was set to 10^{-5} eV to guarantee reliable outcomes. To investigate the electronic structure of Ti_2InB_2 (001) and (010) surfaces, a slab model consisting of six atomic layers with a 3×3 supercell was constructed. These atomic layers were separated from adjacent layers by a vacuum thickness of 20 Å along the Z direction (refer to **Figure S1**). To assess the impact of the indium vacancy on the electronic structure of Ti_2InB_2 (001), calculations were performed to determine the charge density and densities of states (DOS) of V_{In}-Ti_2InB_2 (001). These calculations aimed to evaluate any changes induced by the presence of the indium vacancy.

The adsorption energies (E_b) of Li atoms on various surfaces were determined using the following equation:

$$E_b = E_{Li@surf} - E_{surf} - E_{Li} \quad (1)$$

where $E_{Li@surf}$, E_{surf} and E_{Li} are the calculated energies of Li@surface, bare surface and a Li atom in the most stable Li metal phase, respectively.

The climbing-image nudged elastic band (CI-NEB) approach was employed to calculate the energy barrier associated with the migration of Li^+ ions[5]. This method is commonly used for studying reaction pathways and transition states. In addition, charge density difference analysis and Bader charge analysis were conducted to investigate the charge distributions within the surface structures[6]. These analyses provide insights into the redistribution of charges and allow for a better understanding of the electronic properties and interactions involved.

The detailed calculation method of theoretical capacity: A 3 × 3 supercell of V_{In}-Ti_2InB_2 and Ti_2InB_2 monolayer were constructed. These atomic layers were separated from adjacent layers by a vacuum thickness of 50 Å along the Z direction (**Figure S12(a-d)**). According to our calculated

adsorption energies of a Li atom at different sites on the (010) and (001) surfaces of V_{In}-Ti_2InB_2 and Ti_2InB_2, we adsorb lithium atoms to the site of In vacancy titanium surface of (001) (**Figure S12(e-f)**). The detailed calculation method is: (1) The average adsorption energy, E_{ave}, is defined as

$$E_{ave} = \frac{E_{Li_{x@surface}} - E_{surface} - (x)E_{Li}}{x}$$

where $E_{surface}$ represents the energy of V_{In}-Ti_2InB_2 and Ti_2InB_2 monolayer, $E_{Li_{x@surface}}$ represents the energy of x Li atoms adsorbed at the V_{In}-Ti_2InB_2 and Ti_2InB_2 monolayer, E_{Li} represent the energy of each Li atom in bulk, and x is the number of Li atoms adsorbed. When E_{ave} changes from negative to positive, the adsorbed Li atoms gather to form clusters due to the cohesive energy and further adsorption will stop. (2) The theoretical capacity, C, is defined as

$$C = \frac{xnF}{M_{surface} + xM_{Li}}$$

where x represents the maximum number of atoms adsorbed, n represents the valence of the Li atoms, F represents the Faraday constant (26,801 mAh g^{-1}), and Li atoms to the $M_{surface}$ and M_{Li} represent respective molar weights of V_{In}-Ti_2InB_2/Ti_2InB_2 monolayer and Li.

Supplementary Figures

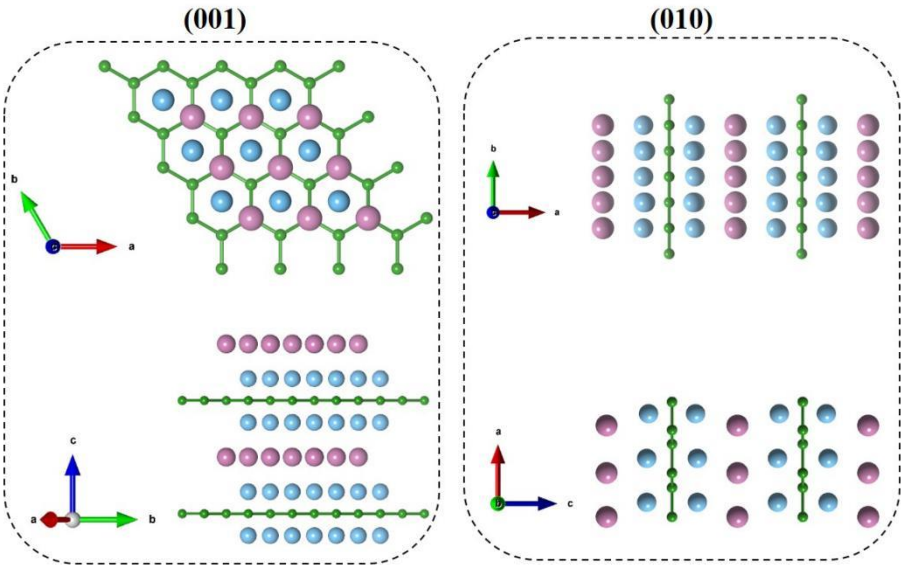

Figure S1: Illustration of slab models of Ti_2InB_2 (001) and (010) surface.

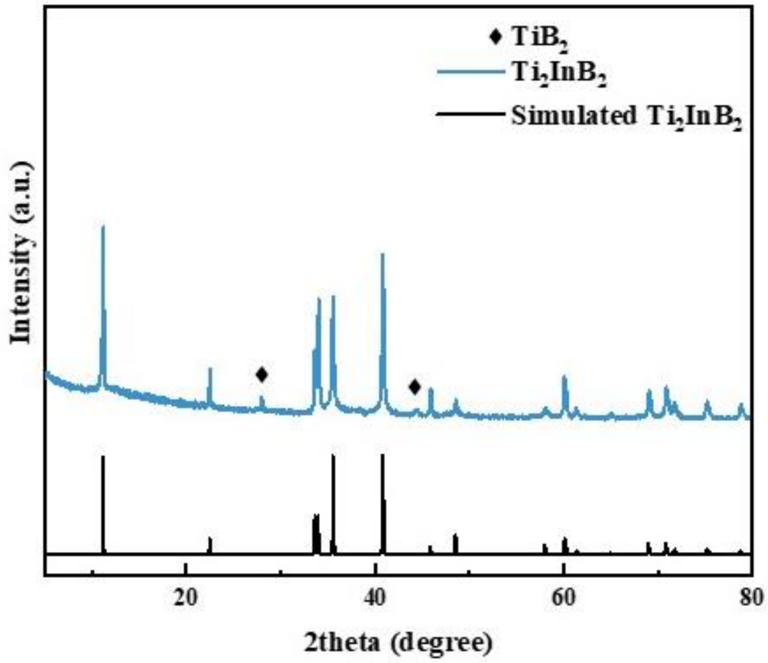

Figure S2: XRD pattern of Ti_2InB_2 MAB phase after HCl etching in comparison with simulated one.

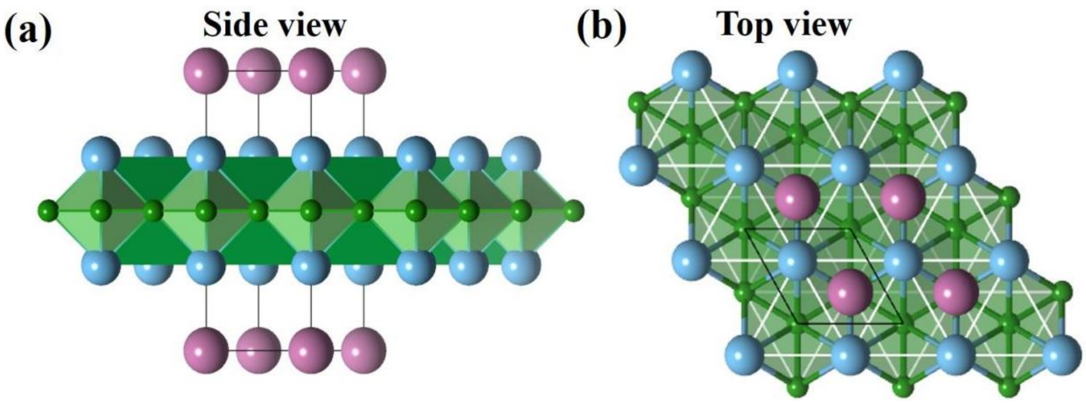

Figure S3: (a) Side and (c) Top views of the crystal structure mode of *h*-MAB phase Ti_2InB_2.

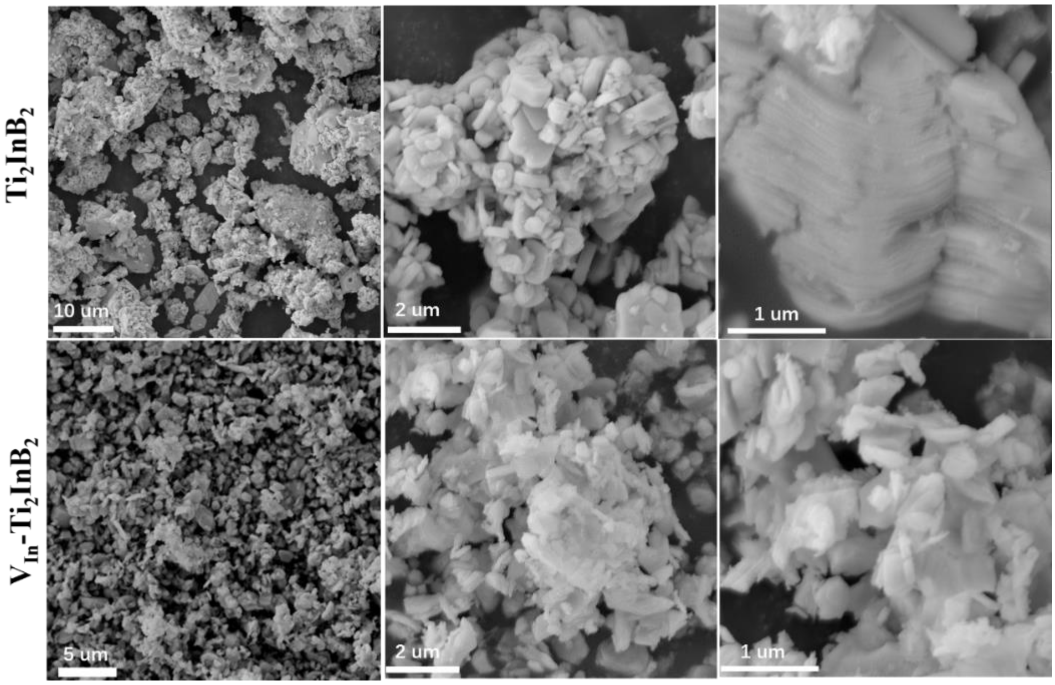

Figure S4: SEM images of Ti_2InB_2 and V_{In}-Ti_2InB_2 *h*-MAB phases with different magnifications.

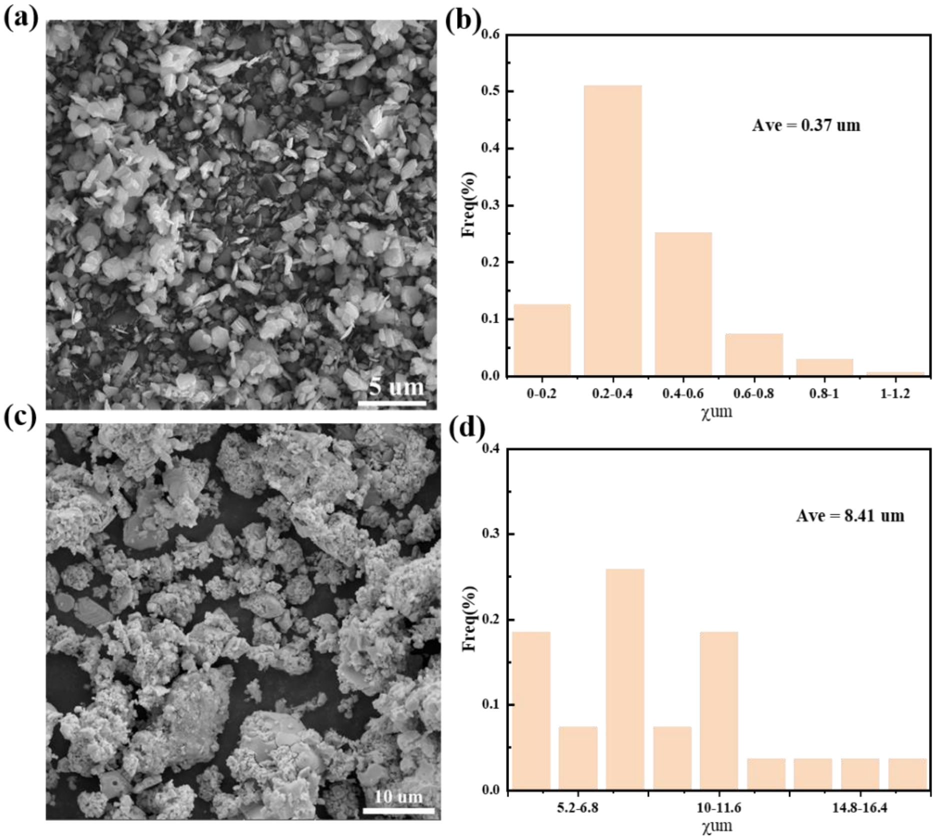

Figure S5: (a) SEM image and (b) particle size distribution of V_{In}-Ti_2InB_2 phase after ball

milling; (c) SEM image and (d) particle size distribution of pristine Ti_2InB_2.

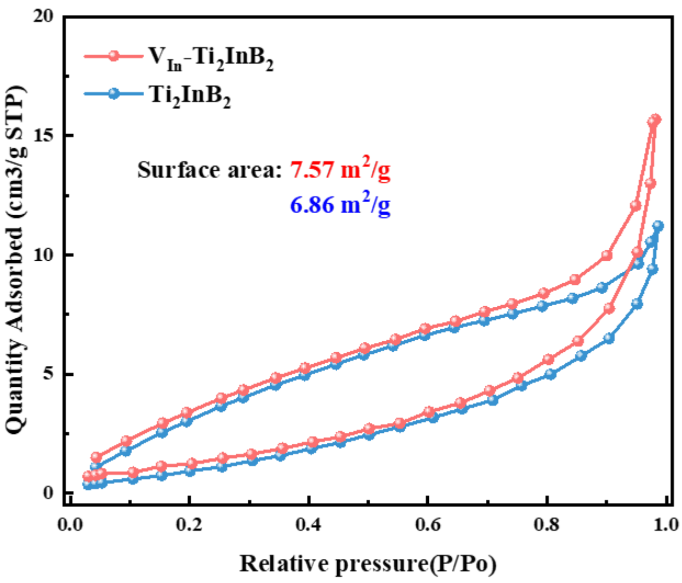

Figure S6: The N_2 adsorption-desorption isotherms curves of V_{In}-Ti_2InB_2 and Ti_2InB_2 phases.

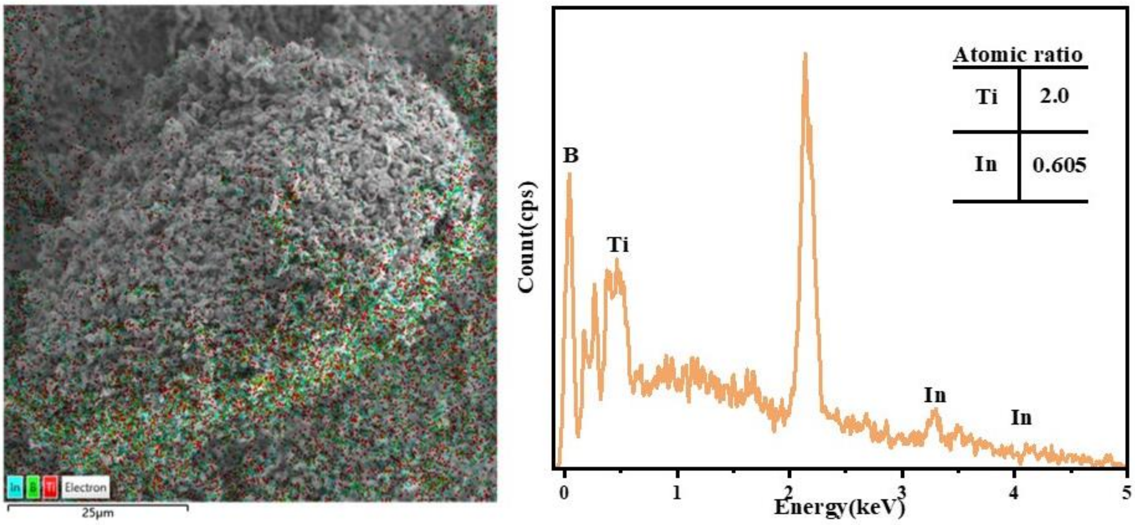

Figure S7: EDS elemental mapping images of V_{In}-Ti_2InB_2.

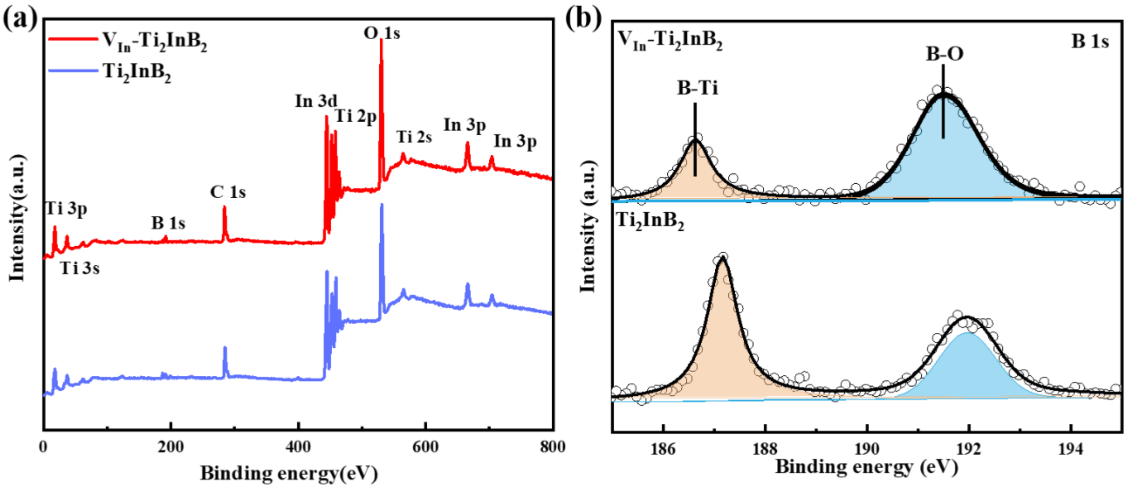

Figure S8: (a) the XPS full survey spectrums and (b) The high-resolution XPS spectra of B 1s for the V_{In}-Ti_2InB_2 and Ti_2InB_2 samples.

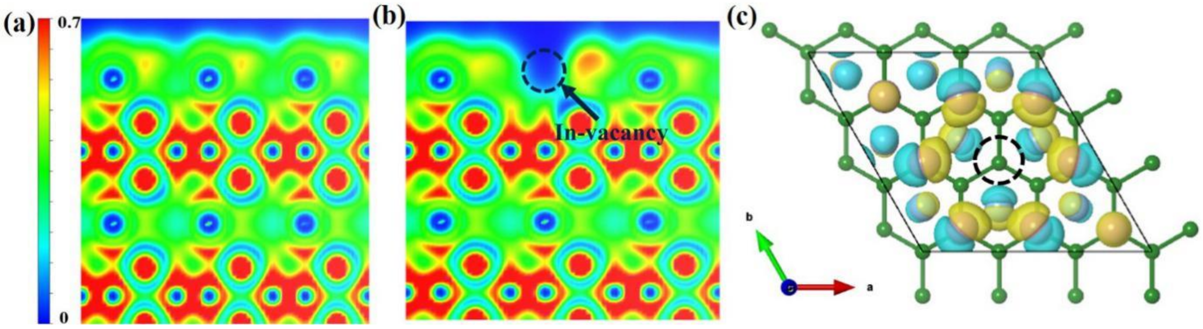

Figure S9: Calculated electron localization functions of (a) Ti_2InB_2 and (b) V_{In}-Ti_2InB_2; (c) charge density difference of V_{In}-Ti_2InB_2 structures. The yellow and cyan surfaces in (c) indicate the charge gain and lost regions, respectively (with isovalue of 0.005).

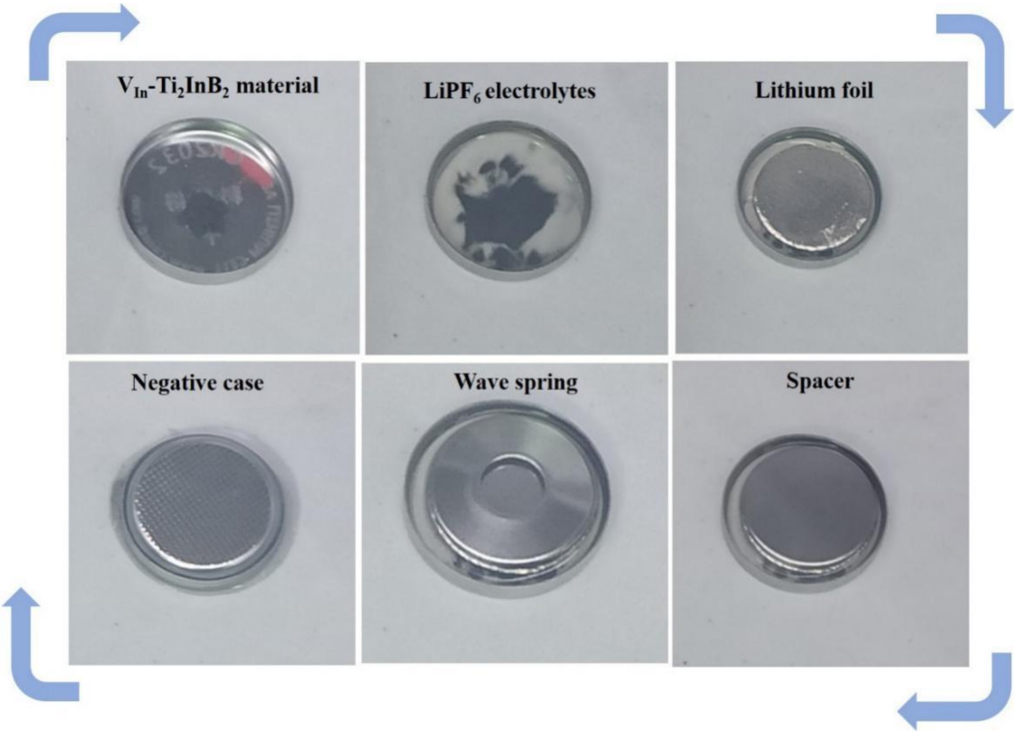

Figure S10: Photos of lithium-ion battery assembled with pure V_{In}-Ti_2InB_2 powder as electrode material.

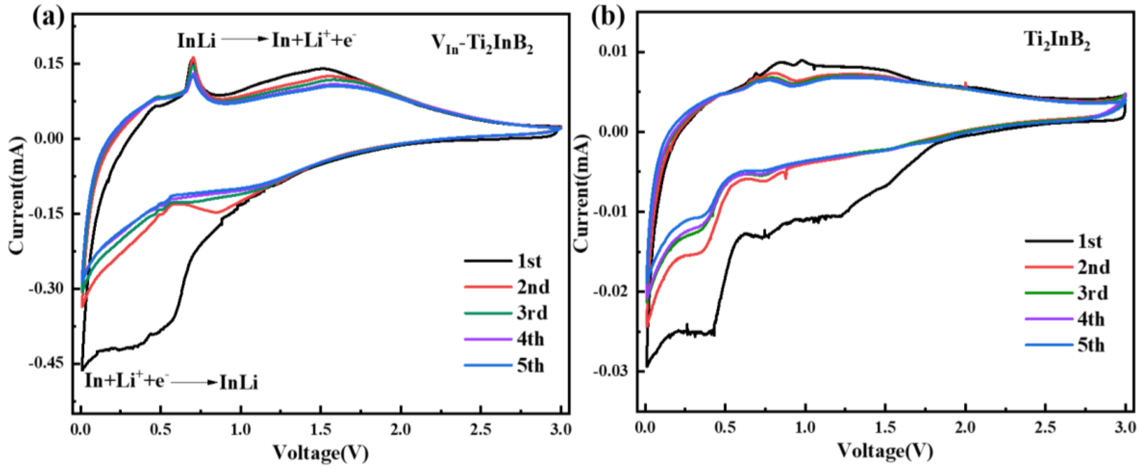

Figure S11: CV curves of V_{In}-Ti_2InB_2 and Ti_2InB_2 electrodes at a scan rate of 0.1 mV s^{-1}.

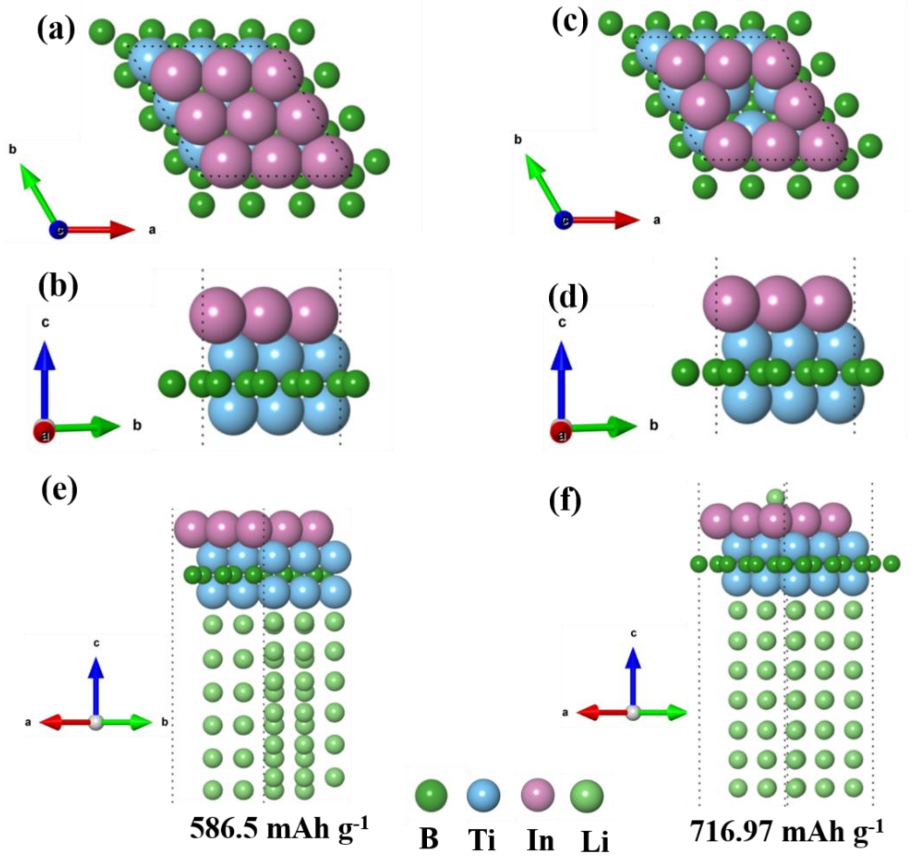

Figure S12: Optimized structures of (a-b) Ti_2InB_2 and (c-d) V_{In}-Ti_2InB_2 monolayers; stable adsorption configurations and capacities of Li^+ on (e) Ti_2InB_2 and (f) V_{In}-Ti_2InB_2 monolayers.

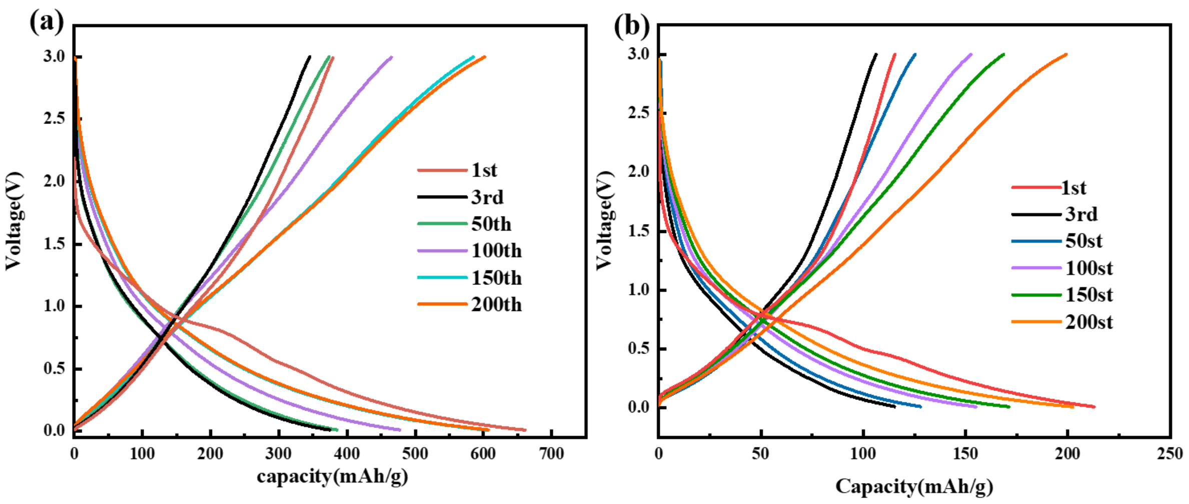

Figure S13: (a) Charge/discharge profiles of V_{In}-Ti_2InB_2 and (b) pristine Ti_2InB_2 anodes at a current density of 0.1 A g^{-1}.

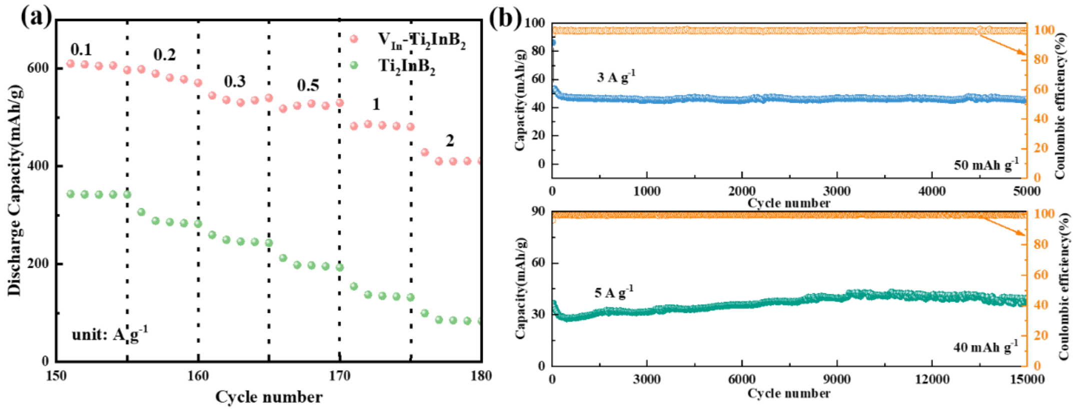

Figure S14: (a) Rate performance of V_{In}-Ti_2InB_2 and pristine Ti_2InB_2 anodes after long cycling at a current density of 0.1A g^{-1}; (b) Long-term cyclic performance of Ti_2InB_2 electrodes at current densities of 3 A g^{-1} and 5 A g^{-1}.

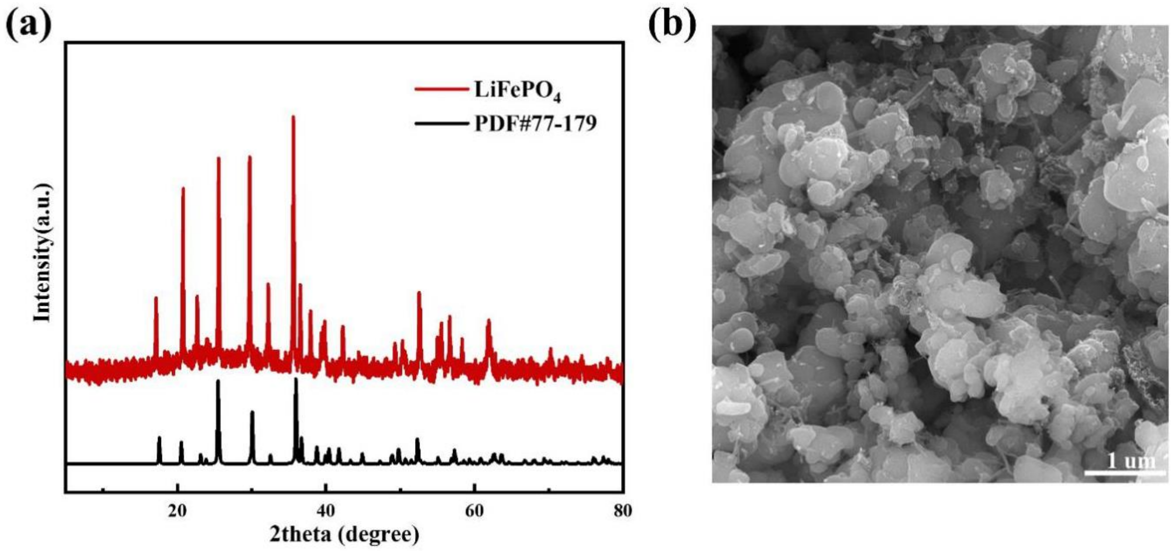

Figure S15: (a) XRD pattern and (b) SEM image of $LiFePO_4$.

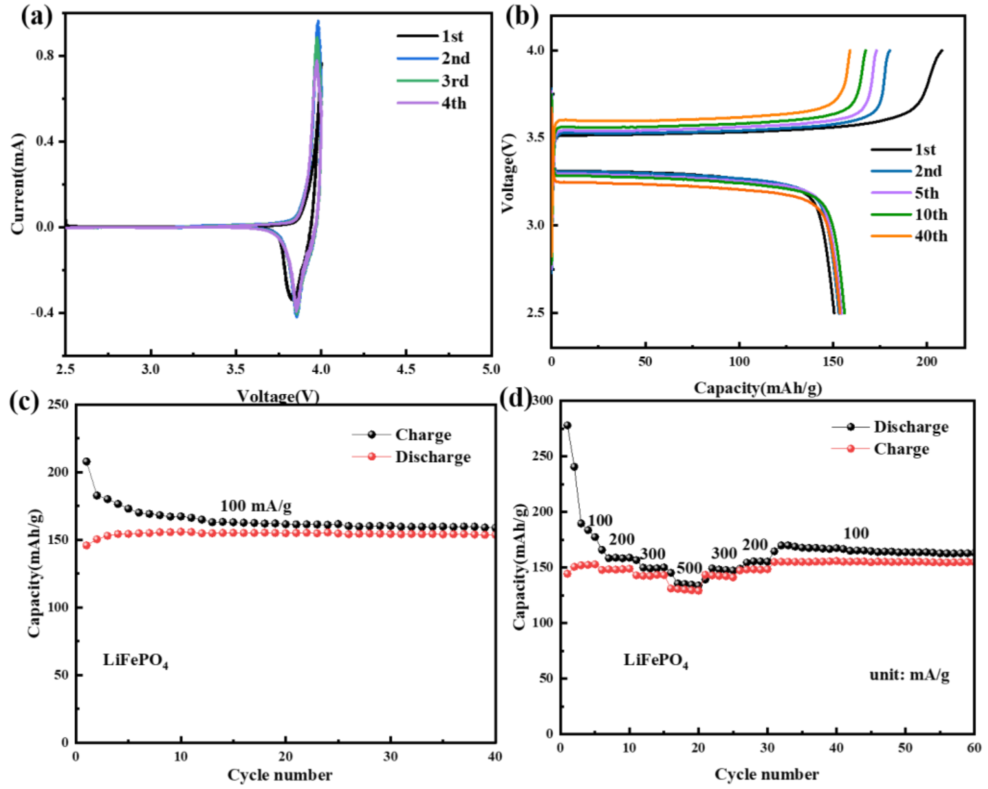

Figure S16:(a) CV curves; (b) GCD profiles at 100 mA g^{-1} ;(c) long cycle and (d) rate performance of LFP electrode.

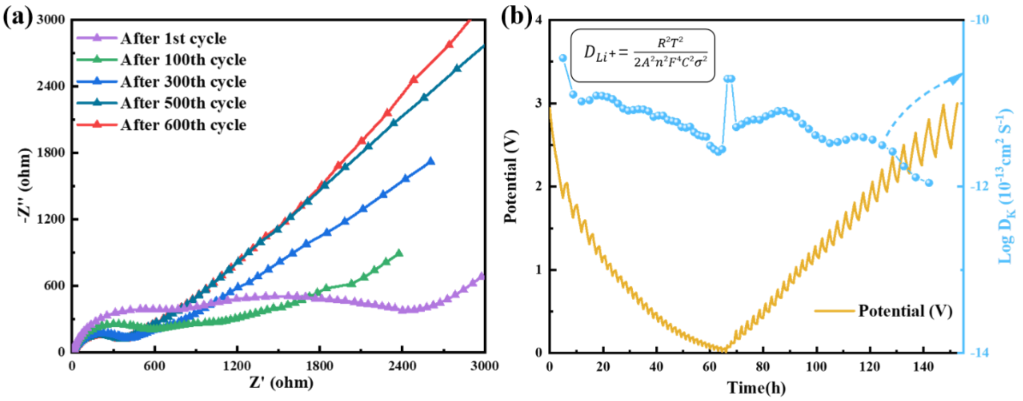

Figure S17： (a) EIS measurements of the pristine Ti_2InB_2 electrode after different cycles; (b) GITT results of V_{In}-Ti_2InB_2 electrode treated at 50 mA g^{-1}.

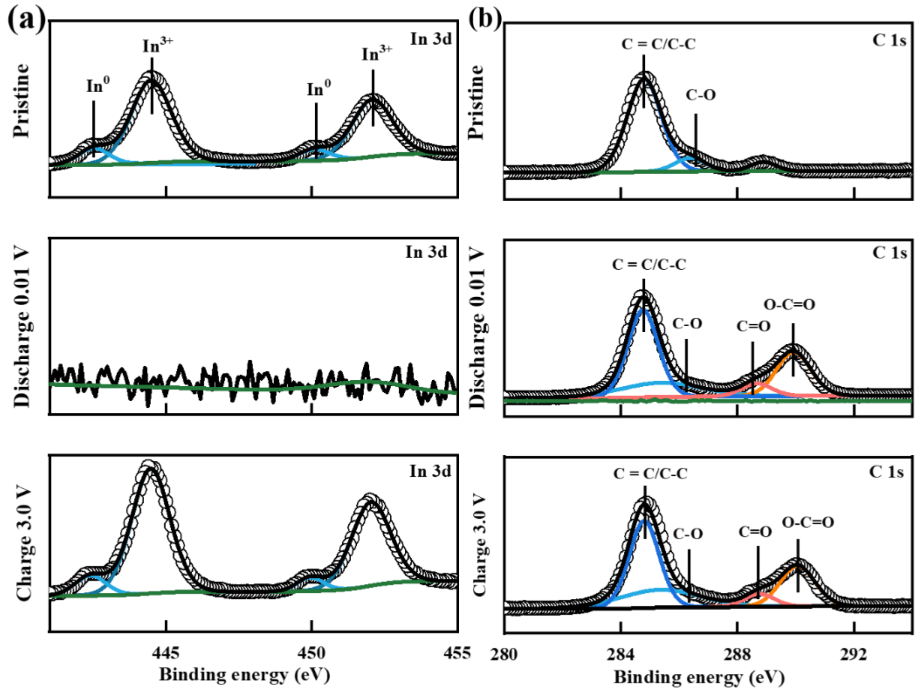

Figure S18: (a) Ex-situ XPS In 3d and (b) C 1s spectra of V_{In}-Ti_2InB_2 electrode during the 1st charging/discharging, at 100 mA g^{-1} current density.

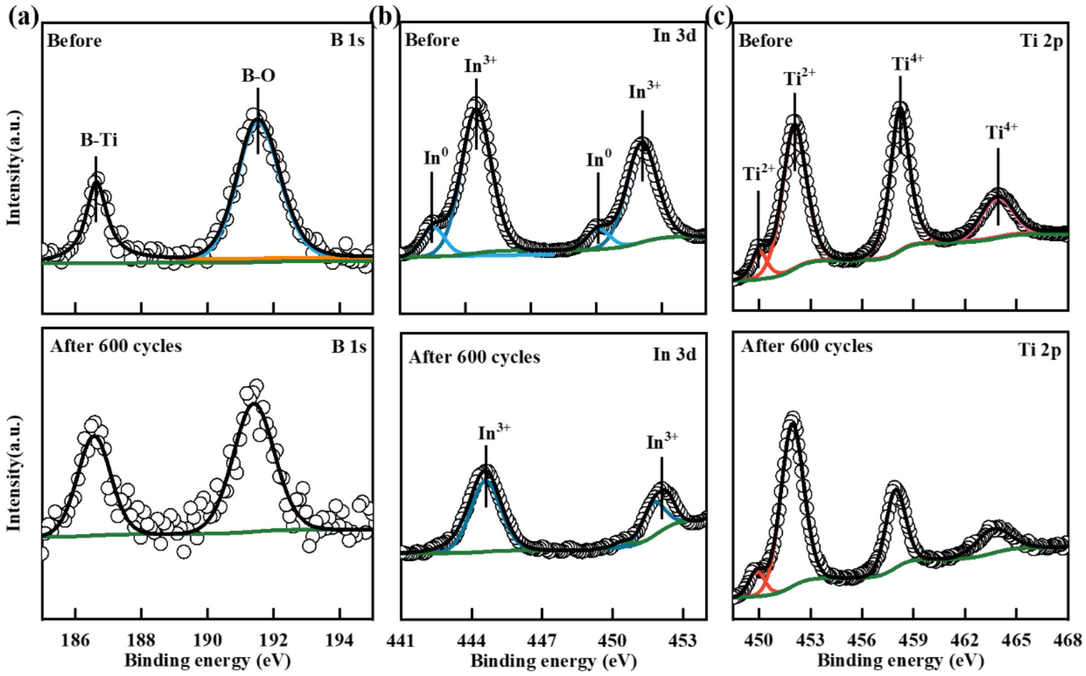

Figure S19: (a) XPS spectra for B 1s; (b) In 3d and (c) Ti 2p region of V_{In}-Ti_2InB_2 before and after 600 cycles at 1 A g^{-1}.

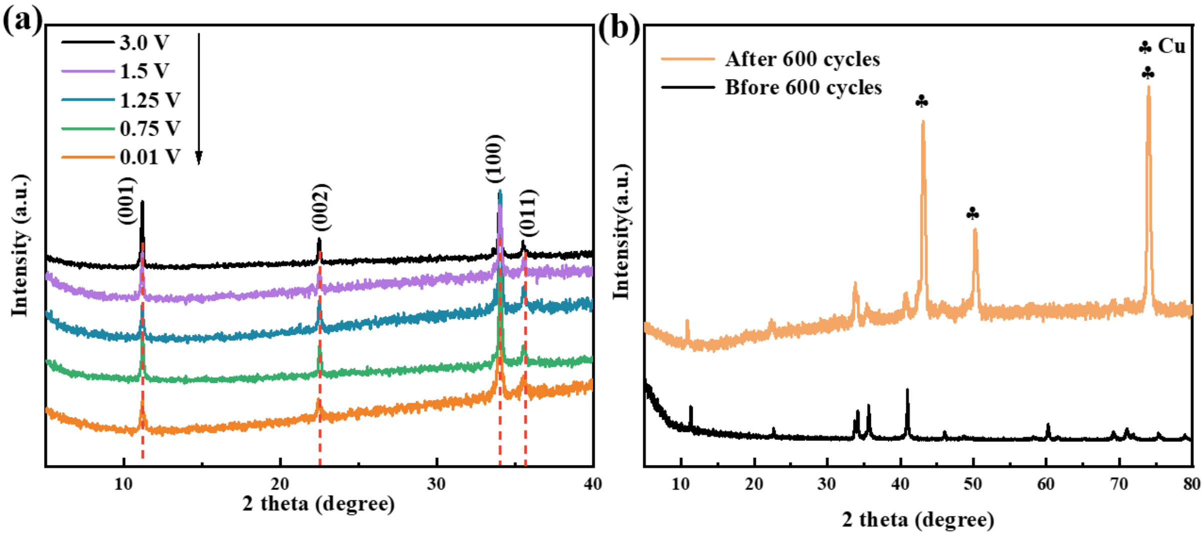

Figure S20: (a) Ex-situ XRD patterns at different voltages of the second cycle; (b) XRD patterns of V_{In}-Ti_2InB_2 electrode before and after 600 cycles at 1A g^{-1}.

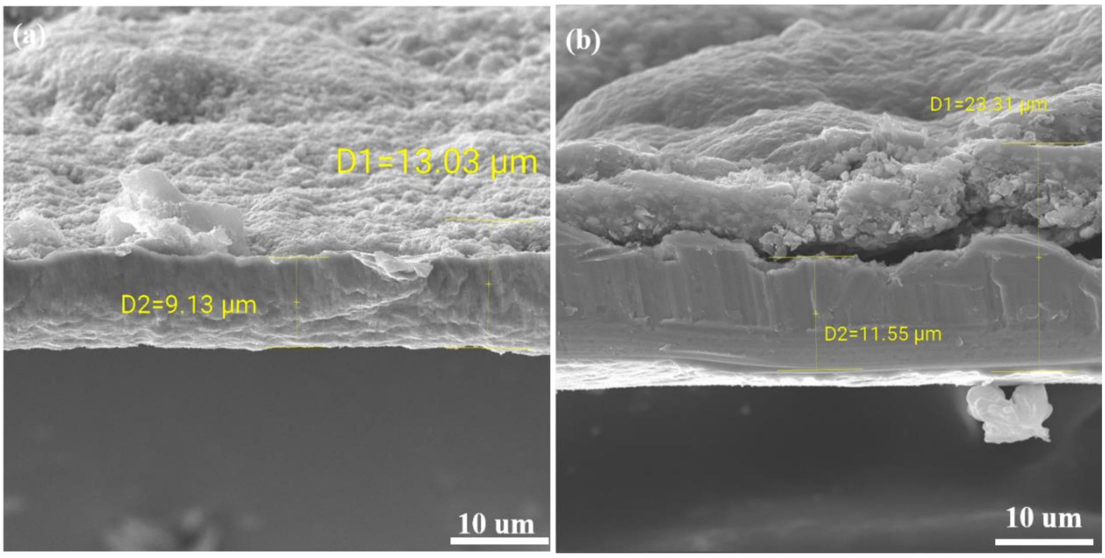

Figure S21: Cross-section SEM views of V_{In}-Ti_2InB_2 electrode (a) before and (b)after 600 cycles at a current density of 1 A g^{-1}.

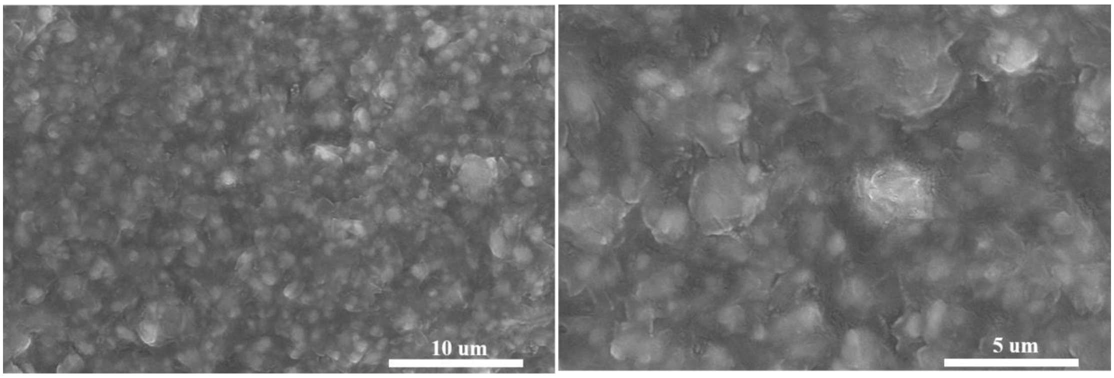

Figure S22: Top view of SEM images of V_{In}-Ti_2InB_2 electrode after 600 cycles at 1 A g^{-1}.

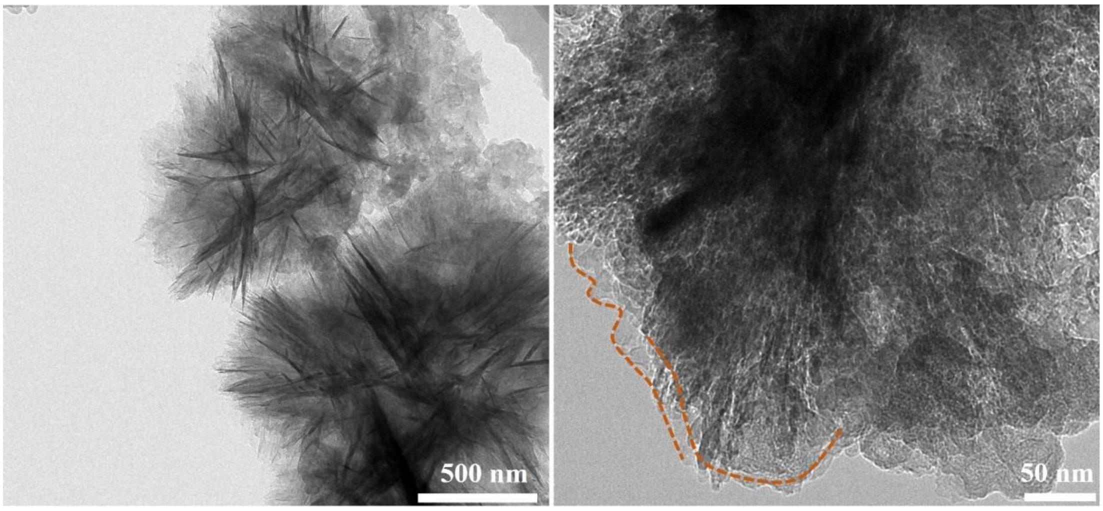

Figure S23: TEM images of V_{In}-Ti_2InB_2 electrode after 600 cycles at 1 A g^{-1}.

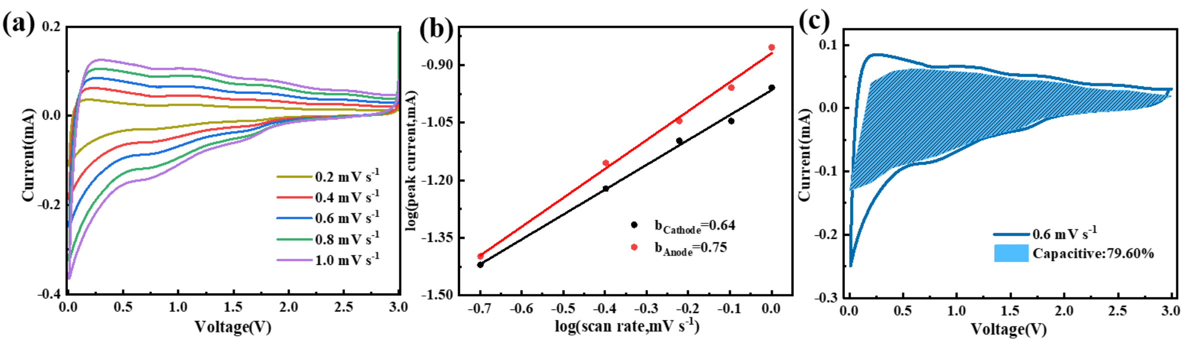

Figure S24: (a) CV curves of pre-cycling V_{In}-Ti_2InB_2 electrode at different scan rates; (b) Log (*i*)

vs. log (v) plots calculated from CV curves of pre-cycling V_{In}-Ti_2InB_2 electrode; (c) The CV curve of pre-cycling V_{In}-Ti_2InB_2 electrode with capacitive contribution in the shaded region at a scan rate of 0.6 mV s^{-1}.

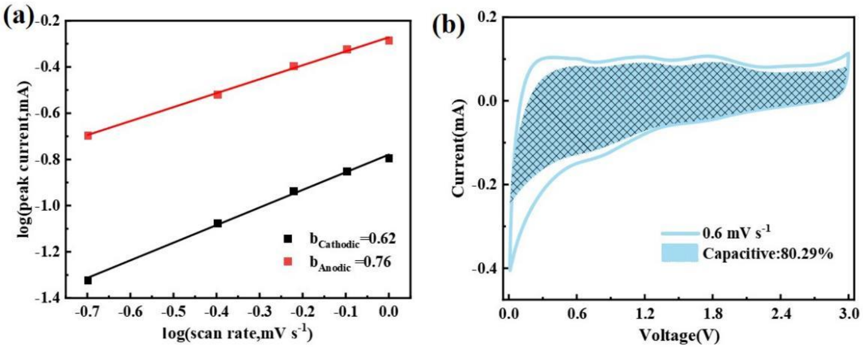

Figure S25: (a) Log (i) vs. log (v) plots calculated from CV curves of V_{In}-Ti_2InB_2 electrode after 600 cycles and (b) The CV curve of V_{In}-Ti_2InB_2 electrode after 600 cycles with capacitive contribution in the shaded region at a scan rate of 0.6 mV s^{-1}.

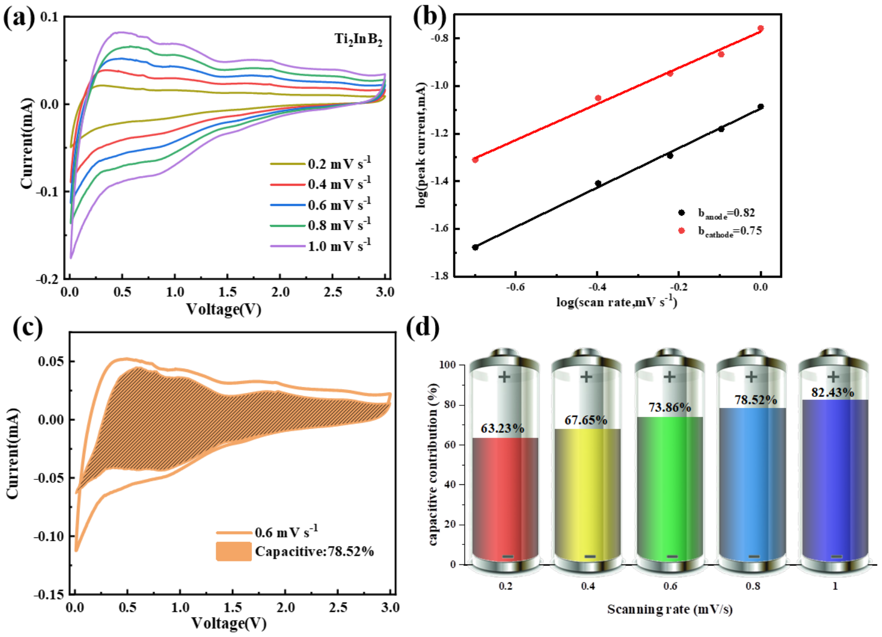

Figure S26: (a) CV curves of pre-cycling Ti_2InB_2 electrode at different scan rates; (b) Log (i) vs.

log (v) plots calculated from CV curves of pre-cycling Ti_2InB_2 electrode; (c) The CV curve of pre-cycling Ti_2InB_2 electrode with capacitive contribution in the shaded region at a scan rate of 0.6 mV s^{-1}.

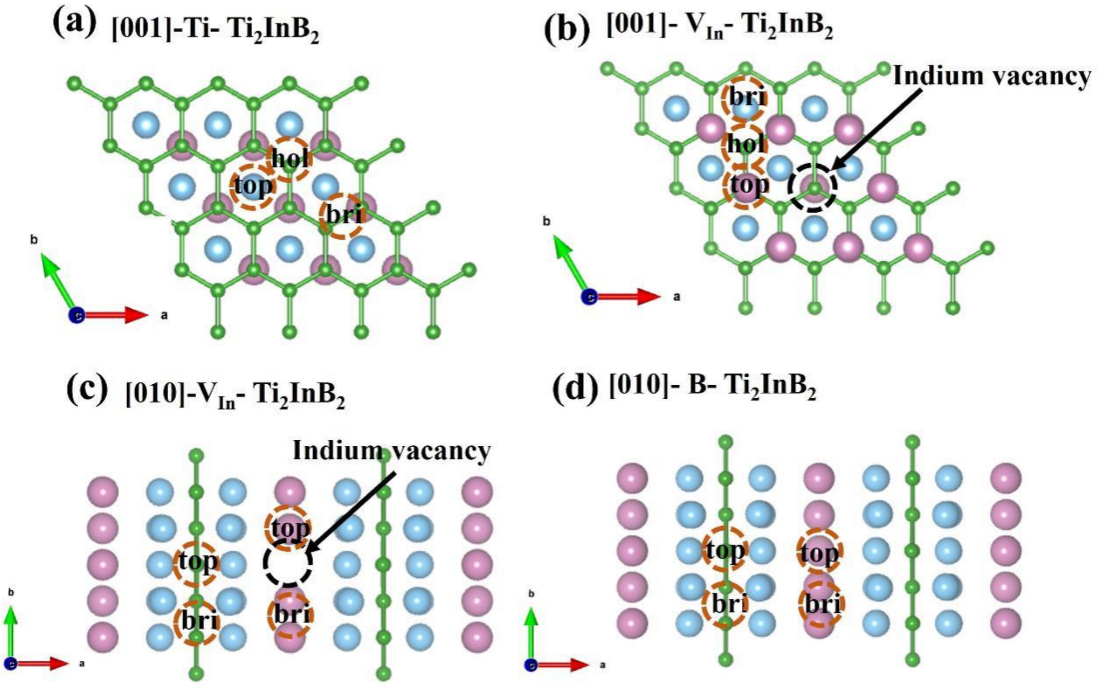

Figure S27: The possible adsorption sites for Li atom at (a and b) (001) and (c and d) (010) surfaces of (a and d) pristine Ti_2InB_2 and (b and c) V_{In}-Ti_2InB_2.

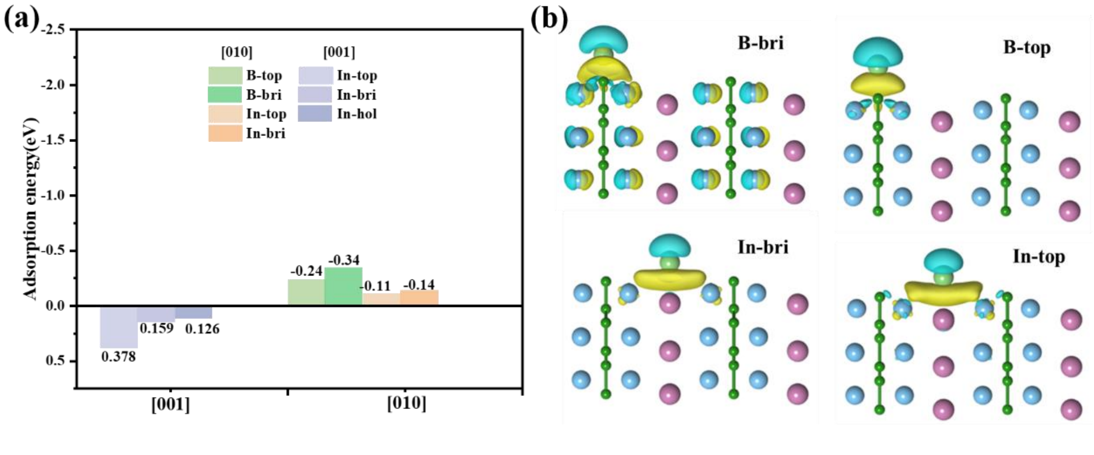

Figure S28: (a) Calculated adsorption energies of Li^+ ion at different sites on the (010) and (001) surfaces of pristine Ti_2InB_2; (b) The calculation results of charge density difference on the (010)

surface of pristine Ti_2InB_2. The yellow and cyan surfaces indicate the charge gain and lost regions, respectively (isovalue, 0.0015).

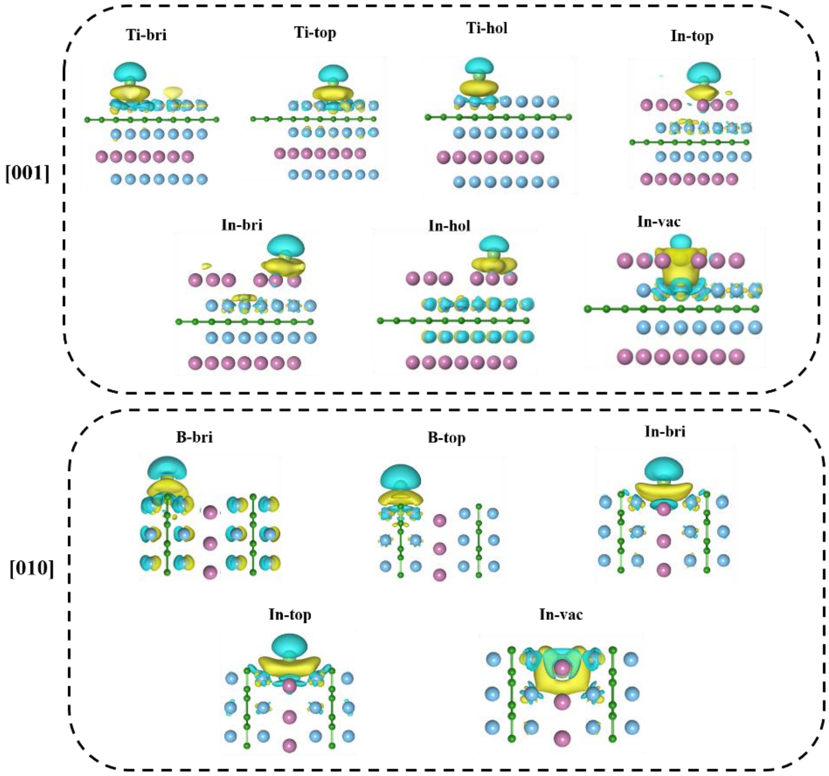

Figure S29: The calculation results of charge density difference on the (001) and (010) surface of V_{In}-Ti_2InB_2. The yellow and cyan surfaces indicate the charge gain and lost regions, respectively (isovalue, 0.0015).

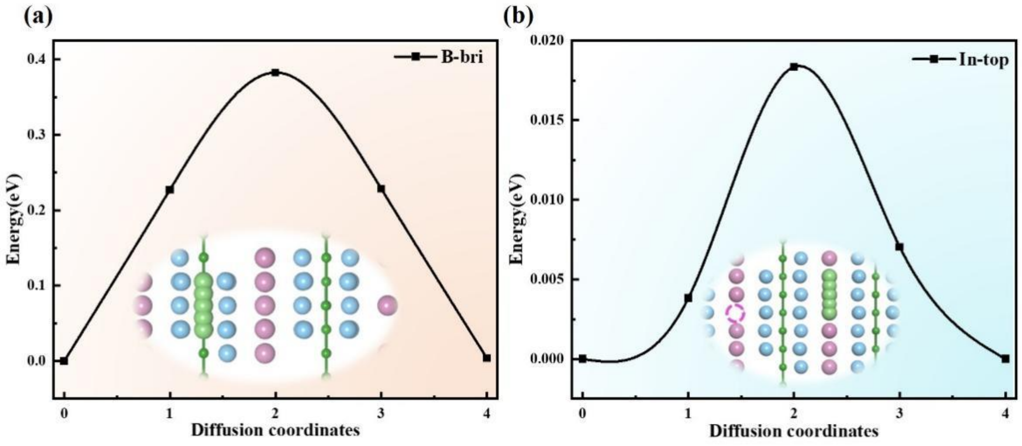

Figure S30: Calculated energy barriers for Li migration along the paths of (a) B-bri to B-bri and (b) In-top to In-top on the (010) surface of V_{In}-Ti_2InB_2.

Supplementary Table

Table S1. Element contents of pristine Ti_2InB_2 and V_{In}-Ti_2InB_2 obtained by XPS analysis.

Materials	Element (at %)				
	C	O	Ti	In	B
**Ti_2InB_2**	32.09	20.2	19.29	8.83	19.59
**V_{In}-Ti_2InB_2**	24.24	34.18	18.51	5.11	17.96

Table S2. Comparison of the electrochemical performances of V_{In}-Ti_2InB_2 anode with those reported MAX/MAB phase anode for LIBs.

Materials	Max Capacity (mAh g^{-1})	Rate capacity (mAh g^{-1})	Cycling Performance			Reference
			Rate	Cycle Number	Capacity (mAh g^{-1})	
Ni_2ZnB	90 (0.1 A/g)	70 (0.3 A/g)	0.3 A/g	400	70	[16]
Nb_2SnC	234 (0.05 A/g)	151 (0.5 A/g)	0.5 A/g	1500	150	[15]
Ti_3SiC_2	350 (0.05 A/g)	85 (2 A/g)	1 A/g	3000	180	[10]
Ti_2SC	350 (0.4 A/g)	140 (4 A/g)	4 A/g	3000	130	[8]
V_2SnC	490 (0.05 A/g)	100 (5 A/g)	1 A/g	1000	260	[14]
$Ti_3Si_{0.75}Al_{0.25}C_2$	350 (0.2 A/g)	150 (8 A/g)	8 A/g	200	150	[11]
Ti_2SC_2	120 (0.05 A/g)	44 (2 A/g)	0.4 A/g	1000	350/	[9]
Ti_2SnC_2@CNF	500 (0.1 A/g)	320 (2 A/g)	1 A/g	500	367.5	[13]
Ti_2SnC_2	735 (0.05 A/g)	214 (2 A/g)	0.4 A/g	1000	400	[14]

V_{In}-Ti_2InB_2	600 (0.1 A/g)	100 (5 A/g)	1 A/g<br>5 A/g	**800**<br>**5000**	**400**<br>**92**	**This work**

Referencens

[1] J. Wang, T.-N. Ye, Y. Gong, J. Wu, N. Miao, T. Tada, H. Hosono, Nat Commun 2019, 10, 2284.

[2] G. Kresse, J. Furthmüller, Computational Materials Science 1996, 6, 15–50.

[3] P. E. Blöchl, Phys. Rev. B 1994, 50, 17953–17979.

[4] J. P. Perdew, K. Burke, M. Ernzerhof, Phys. Rev. Lett. 1996, 77, 3865–3868.

[4] G. Mills, H. Jónsson, Phys. Rev. Lett. 1994, 72, 1124–1127.

[5] W. Tang, E. Sanville, G. Henkelman, J. Phys.: Condens. Matter 2009, 21, 084204.
